# Rlip Depletion Suppresses Growth of Breast Cancer

**DOI:** 10.3390/cancers12061446

**Published:** 2020-06-02

**Authors:** Chhanda Bose, Sushma Yadav, Sharad S. Singhal, Jyotsana Singhal, Ashly Hindle, Jihyun Lee, Naga K. S. Cheedella, Shabnam Rehman, Rakhshanda Layeequr Rahman, Catherine Jones, Meenakshi Darden, Philip T. Palade, David Berz, Sharda P. Singh, Sanjay Awasthi

**Affiliations:** 1 Department of Internal Medicine, Division of Hematology & Oncology, Texas Tech University Health Sciences Center, Lubbock, TX 79430, USA; chhanda.bose@ttuhsc.edu (C.B.); ashly.hindle@ttuhsc.edu (A.H.); jihyun.lee@ttuhsc.edu (J.L.); Naga.Cheedella@ttuhsc.edu (N.K.S.C.); Shabnam.Rehman@ttuhsc.edu (S.R.); catherine.jones@ttuhsc.edu (C.J.); 2 Department of Translational Research and Cellular Therapeutics, City of Hope Comprehensive Cancer Center, Duarte, CA 910102, USA; syadav@coh.org; 3 Department of Medical Oncology and Therapeutic Research, City of Hope Comprehensive Cancer Center, Duarte, CA 910102, USA; ssinghal@coh.org (S.S.S.); jsinghal@COH.org (J.S.); 4 University Medical Center Health System, UMC Cancer Center, Lubbock, TX 79415, USA; rakhshanda.rahman@ttuhsc.edu; 5 Department of Surgery, Texas Tech University Health Sciences Center, Lubbock, TX 79415, USA; 6 McGovern Medical School of the University of Texas Health Sciences Center, Houston, TX 77030, USA; meenakshi.awasthi@gmail.com; 7 Department of Pharmacology and Toxicology, University of Arkansas for Medical Sciences, Little Rock, AR 72205, USA; ppalade@uams.edu; 8 Beverly Hills Cancer Center, Los Angeles, CA 90211, USA; dberz@bhcancercenter.com

**Keywords:** breast cancer, Rlip76, RalBP1, endocytosis, apoptosis

## Abstract

RLIP76 (RAL-binding protein-1, Rlip) is a stress-protective mercapturic-acid-pathway transporter protein that also plays a key role in regulating clathrin-dependent endocytosis as a Ral effector. Targeted inhibition or depletion of Rlip causes regression of xenografts of many cancers and is capable of abrogating tumor formation in p53-null mice. This is associated with the reversion of the abnormal methylomic profile of p53-null mice to wild-type. In a query of The Cancer Genome Atlas (TCGA) databases, we found that Rlip expression was associated with poor survival and with significant differences in the frequencies of *PIK3CA* mutation, *MYC* amplification, and *CDKN2A/B* deletion, which were the most commonly mutated, amplified, and deleted genes, respectively, among TCGA breast cancer patients. We conducted the present study to further examine the effects of Rlip inhibition and to evaluate the in vitro and in vivo efficacy in breast cancer. Using immunogold electron microscopy, we found that plasma-membrane Rlip was accessible to cell-surface antibodies in the MCF7 (ER+) breast cancer cell line. Rlip depletion resulted in decreased survival of MCF7 and MDA-MB-231 cells and increased terminal deoxynucleotidyl transferase dUTP nick end labeling (TUNEL) positivity and DNA laddering, indicating apoptotic cell death. Additionally, in vitro knockdown of Rlip inhibited EGF endocytosis and WNT/MAPK signaling. Xenograft studies in nude mice showed regression of breast cancer via antisense-mediated depletion of Rlip mRNA as well as by anti-Rlip antibody. Finally, knockdown of Rlip by antisense locked nucleic acid oligonucleotides increased markers for apoptotic signaling and decreased markers for proliferation, angiogenesis, and cell cycling in MCF7 and MDA-MB-231luc xenografts. Our findings validate Rlip as an attractive target in breast cancer.

## 1. Introduction

In the U.S., approximately one in eight women will develop invasive breast cancer during their lifetime, with an estimated 42,000 deaths in 2019 [1]. The 20-year overall survival (OS) of patients diagnosed with localized disease or one to three nodal metastases is approximately 35%, a rate that is unaffected by surgery due to undetected distant metastases [2,3,4]. Breast cancer is generally responsive to chemotherapy [5,6], but inevitable de novo or acquired resistance to chemotherapy [7,8,9,10,11], estrogen receptor blockers/estrogen synthesis inhibitors [12], kinase inhibitor therapy [13], and radiation [14,15,16] has been a major impetus to identify resistance mechanisms and to develop effective targeted therapies [17,18,19]. Secreted growth factors and signaling proteins such as EGF, TGF, and WNT mediate multifactorial resistance to a broad spectrum of anticancer therapies by protecting cells from apoptosis and promoting the survival of cancer stem cells through regulation of *TP53* [20], *MYC* [12,13], *PI3KCA*, and *AKT1* [21,22]. Multifactorial resistance to apoptosis is also provided by glutathione (GSH), used to detoxify electrophilic (i.e., cyclophosphamide, carboplatin) and oxidative (i.e., doxorubicin, X-irradiation) therapies. Several enzymes that synthesize or utilize GSH are upregulated in breast cancers and have been correlated with drug or radiation resistance [23,24,25]. The ABC transporters MDR1 and MRP1 have been widely studied as mediators of drug resistance via catalyzing the efflux of cytotoxic drugs and their metabolites [7,8,9,10]; however, these transporters have not been able to fully explain the efflux of chemotherapy drugs from cancer cells [26,27].

It is established that the broad-specificity non-ABC-transporter RLIP76 (Rlip) mediates both the removal of toxic cellular metabolic byproducts as GSH conjugates (GS-E) and the efflux of GSH conjugates of drugs including doxorubicin, vinorelbine, and melphalan [10,14,16,28,29,30]. Rlip is a 76 kDa splice variant protein encoded by the human Ral-binding protein-1 (*RALBP1*) gene [31,32]. It is a Ral-regulated component of clathrin-dependent endocytosis (CDE) that is bound to the AP2 clathrin adaptor protein and to other endocytic components, including POB1 [32,33]. The deficiency of Rlip mutants in their ability to carry out CDE has been shown to correlate with their deficiency in drug efflux activity, indicating that the function of Rlip as an ATP-dependent efflux transporter is coupled with its function as the rate-determining ATPase in endocytosis [34]. The blocking of both drug efflux and receptor internalization following treatment with an anti-Rlip antibody also supports this [10,26,35]. This is important because CDE regulates proliferative signaling via internalization of receptor–ligand complexes from the plasma membrane [34,36]. Thus, Rlip deficiency inhibits downstream proliferation, growth, and survival signaling pathways by interfering with growth factor and peptide hormone signaling. It also promotes apoptosis due to accumulation of toxic mercapturic acid precursors. This dual mechanism of action of Rlip inhibition predicts potent anticancer effects, substantiated in several studies showing that targeted depletion or inhibition of Rlip causes regression of melanoma, neuroblastoma, and carcinomas of the lung, colon, kidney, prostate, and pancreas in mouse models [27,37,38,39,40,41].

Most remarkably, Rlip is likely an existential requirement of many cancers, as supported by the facts that Rlip-knockout mice are highly resistant to carcinogenesis caused by benzo[a]pyrene [34] and that antisense-mediated depletion of Rlip completely abrogates the development of cancer in p53-null mice, which uniformly die from cancer [42]. This latter effect is accompanied by a normalization towards wild-type of the aberrations in promoter CpG island methylation and the corresponding transcriptome that arise with age in the p53-null mice. This is highly relevant to breast cancer because p53 mutations are among the most frequent genetic alterations in this cancer.

Also importantly, CDE plays a crucial role in breast cancer by regulating proliferative EGF, insulin, and WNT signaling. These observations have led us to consider that the existential necessity of Rlip also pertains to breast cancer [42]. In a query of The Cancer Genome Atlas (TCGA) database, we found that *PIK3CA* was the most frequently mutated gene in breast cancer, while *MYC* and *CDKN2A/B* were the genes most frequently associated with amplifications and deletions, respectively. Interestingly, Rlip expression was associated with poor survival and with significant differences in the frequencies of *PIK3CA* mutation, *MYC* amplification, and *CDKN2A/B* deletion (Table 1 and Table 2). In order to further study Rlip as a therapeutic target for breast cancer, we evaluated the in vitro and in vivo efficacy and molecular mechanisms of several methods of Rlip inhibition. Our results show that Rlip inhibition exerted potent anticancer effects in preclinical models of both ER+ and triple negative breast cancer. These findings are supported by a number of mechanistic studies showing an array of molecular pharmacodynamic effects on markers of several cancer hallmarks including endocytosis, proliferation, metastasis, and angiogenesis. We concluded that Rlip is a promising target for breast cancer.

## 2. Results

### 2.1. RALBP1 Gene Expression in Human Breast Cancer

Genomic mutation, amplification, and deletion data were from breast cancer cases in the Molecular Taxonomy of Breast Cancer International Consortium (METABRIC) study dataset [43], as hosted by Memorial Sloan Kettering Cancer Center (MSK) cBioportal.org. [44]. Of the 2509 total cases, mutation data were available in 2509 cases, from which 12,104 mutations were present in 173 genes. Copy number alteration (CNA) data were available in 2173 cases, from which 1,336,083 CNAs were present in 32,949 genes. The most frequent mutations among all cases were in *PIK3CA* (38.9% of cases) and *TP53* (34.4%). The frequency of mutations in these two genes was over three-fold that of the next most frequently mutated gene, *KMT2C* (11.2%) (Supplemental Data S1A). CNAs, consisting of genomic amplifications and deletions, were more frequent overall than mutations. Approximately 35% of all CNAs were present in the top 20 genes, each of which had a frequency of >20%. CNA frequency >10% was present in 325 genes, and >5% in 544 genes. *MYC* was the most frequently amplified gene among all METABRIC cases, followed by *MDM4*, *PIK3C2B*, *ELK4*, and *RAD21*. (Supplemental Data S1A). *CDKN2A*, *CDKN2B*, *MTAP*, *MAP2K4*, and *PTEN* were the most frequently deleted genes (Supplemental Data S1A). Interestingly, *NBEAP1* was the most frequently observed deletion, but is currently designated as a pseudogene. Rlip mutations were entirely absent across all cases, and Rlip gene amplification was rare (0.8%). Deletions of Rlip were even more rare, with only two homozygous deletions (0.09%) across the 2173 cases evaluated for CNAs; thus, Rlip was genomically present in >99.9% of all breast cancers, suggesting that the presence of Rlip functionality may be a fundamental requirement in the oncogenesis of the vast majority of breast cancers. Most remarkably, we found significant differences in the frequencies of mutations and deletions between cases with high versus low Rlip expression, as defined by either RNA-seq or microarray (Supplemental Data S1A–E, S6–S7; Table 1). The differences between Rlip-high, Rlip-low, and Rlip-unaltered were assessed for statistical significance at alpha *p* = 0.05 via unpaired test with Welch correction. For multiple comparisons between the groups, one-way analysis of variance (ANOVA) with the Tukey–Kramer multiple comparison test and Kruskal–Wallis post-hoc test with Dunn’s correction were performed (*p* < 0.05 was considered statistically significant). Comparison of the patterns of gene mutations between Rlip-high and Rlip-low cases from METABRIC revealed that the mutation frequency of *PIK3CA* was significantly lower in the Rlip-high compared with Rlip-low or Rlip-unaltered cases (16.6% vs. 42.5% vs. 45.1%, respectively). Interestingly the mutation frequency of *AHNAK2*, which encodes a nucleoprotein involved in differentiation and metastasis, was decreased in Rlip-high cases, while that of its paralog *AHNAK* was increased (Table 1).

Rlip expression was also correlated with significant differences in overall survival (OS) among several prognostic subsets (log-rank p-values ranging from 0.079 to 0.013; Table 2*).* Highly significant correlations were also found with respect to relapse-free survival (RFS) in several subsets. Several prognostic subgroups, including those with luminal A, luminal B, luminal AR, or Basal 1 histopathology, those having received prior chemotherapy, and those with Grade 3 tumors, all had significantly longer RFS associated with low Rlip expression (*p* < 0.01).

### 2.2. Rlip Is Localized to Cell Membranes

**Figure 1:**
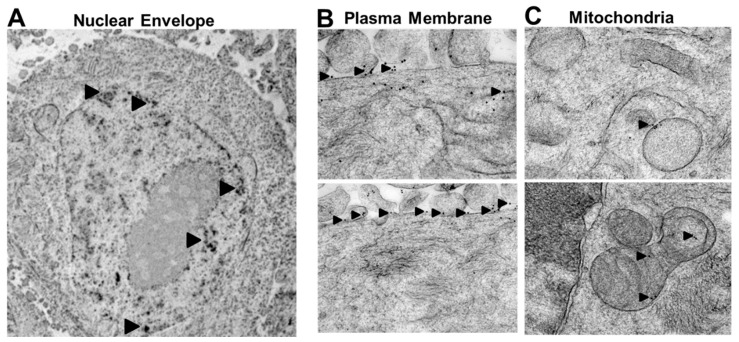
Subcellular localization of Rlip imaged by electron microscopy. Immunogold EM on MCF7 cells was performed as described in the Materials and Methods. Electron microscopy was done on a FEI Tecnai 12 transmission electron microscope equipped with a CCD camera. Black arrow heads point to staining of (**A**) nuclear membrane as well as of the (**B**) plasma membrane and (**C**) mitochondria. Magnification of the image: 11,000×.

Using deletion mutants involving the antennapedia homeodomain-homologous sequences in the N-terminal of Rlip, we identified an internal peptide sequence of Rlip (aa^171–185^) on the cell surface of lung cancer cells [45]. Antibodies targeted to this epitope inhibit the transport activity of Rlip, causing apoptosis and intracellular accumulation of pro-apoptotic alkenals and xenobiotics [46]. In the present study, we used immunogold electron microscopy (City of Hope EM Core facility) to demonstrate that Rlip was indeed present in or adjacent to the plasma membrane, mitochondrial membrane, and nuclear envelope of breast cancer cells (Figure 1A–C). We previously demonstrated the surface expression of RLIP76 by flow cytometry in live breast cancer cells by staining with anti-Rlip IgG (aa^171–185^) [42]. The negative staining of Rlip-KO mouse embryonic fibroblasts (MEFs) was used as a control to test the specificity of Rlip antibodies (Supplemental Data S12). These studies showed that Rlip is widely distributed in cell membranes and that the aa^171–185^ epitope is found on the cell surface of breast cancer cells. These results are consistent with other studies demonstrating the membrane-associated functionality of Rlip and its plasma membrane, nuclear, and mitochondrial localization [34,47,48,49].

### 2.3. Anticancer Effects of Rlip Depletion on Breast Cancer Cell Lines In Vitro

**Figure 2:**
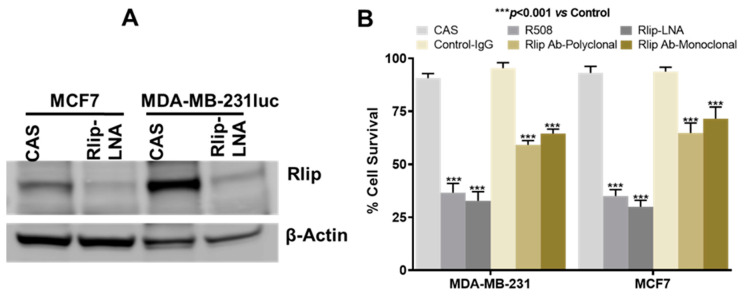
Effects of Rlip depletion on breast cancer cell survival. (**A**) A representative Western blot showing the efficacy of transfection with Rlip-LNA. Cells were transfected for 48 h, and expression level of Rlip was determined using Western blots. β-actin was used as a loading control. (**B**) Effect of Rlip depletion by Rlip antisense and Rlip antibodies on breast cancer cell survival: MCF7 and MDA-MB-231 cells were transfected with Rlip antisense (R508 or LNA) or with Rlip monoclonal (clone RALBP1-A192 from Sigma, St. Louis, MO, USA) or custom polyclonal (Rlip IgG (aa^171–185^) antibodies for 48 h, as described in the Materials and Methods. Following treatment, cells were analyzed by MTT assay. The data were analyzed using two-tailed Student’s *t*-test. A one-way analysis of variance (ANOVA) with post-hoc Tukey’s test was applied to compare all groups. The data presented shows significant (*** *p* < 0.001) cell death after Rlip depletion either by antisense or Rlip antibodies as compared to their corresponding controls. The expressed values are means ± SD. (*n* = 3 independent experiments with eight replicates).

We extensively studied and validated the phosphorothioated Rlip-specific antisense R508 in previous studies for its effects on Rlip depletion, tumor regression, apoptosis, cell cycling, and cell survival [7,27,50,51]. Previously, we demonstrated that R508-mediated Rlip depletion caused a concentration-dependent decrease in proliferation of the MCF7 cell line in culture [52]. Locked nucleic-acid-modified antisense oligonucleotides (LNAs) are third generation antisense molecules with improved pharmacological properties. Therefore, we used Rlip antisense locked nucleic acid (Rlip-LNA; sequence identical to R508), with a control scrambled antisense (CAS), and tested its effects on breast cancer using in vitro and in vivo models. We studied the growth-inhibitory effects of Rlip-LNA on the MCF7 and MDA-MB-231 human breast cancer cell lines. MCF7 cells are estrogen- and progesterone-receptor-positive but HER2-negative (ER+, model for hormone therapy); on the other hand, MDA-MB-231 cells are triple negative (TNBC, model for chemotherapy). Consistent with its predicted high efficiency, Rlip-LNA effectively depleted Rlip protein in cultured MDA-MB-231luc and MCF7 cells (Figure 2A, Supplemental Data S15). Results also suggested that Rlip expression is higher in triple negative MDA-MB-231luc cells when compared with MCF7. We further compared the growth/survival effects of R508, Rlip-LNA, and Rlip antibodies (mono- or polyclonal) on MCF7 and MDA-MB-231 cells. Rlip-LNA resulted in a good breast cancer cell kill rate (29% survival fraction for MCF7 and 32% survival fraction for MDA-MB-231), equivalent to or better than R508 (37% survival fraction for MCF7 and 35% survival fraction for MDA-MB-231, *p* < 0.005) (Figure 2B). Rlip antibody (mono- or polyclonal) treatment inhibited the proliferation of both cell lines, as assessed by cell cytotoxicity assay; however, we found that Rlip-LNA killed breast cancer cells with greater efficiency in vitro (Figure 2B). Rlip depletion with R508 in MCF7 cells also reduced the number of colony-forming units relative to PBS or CAS (Figure S1A).

### 2.4. Rlip-LNA Induces Apoptosis in Breast Cancer Cell Lines In Vitro

**Figure 3:**
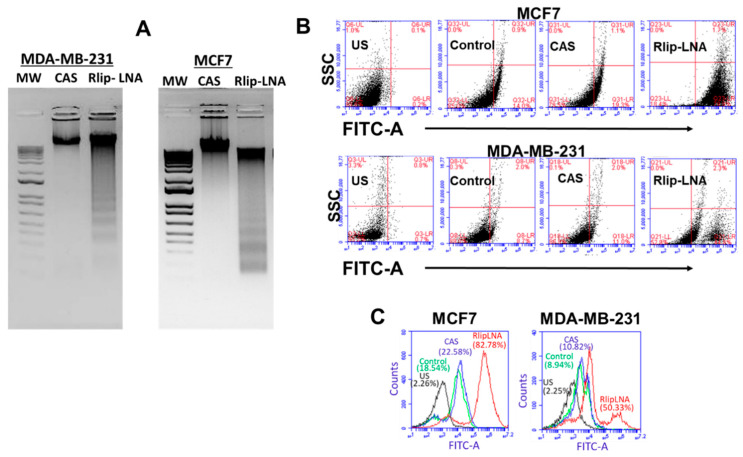
Effect of Rlip-LNA on apoptosis and DNA fragmentation in MDA-MB-231 and MCF7 cells as measured by terminal deoxynucleotidyl transferase dUTP nick end labeling (TUNEL) and DNA laddering assays. (**A**) Apoptosis by DNA laddering. After 48 h of treatment with CAS or Rlip-LNA, DNA was extracted and subjected to agarose-gel electrophoresis in 2% agarose with a 1 kB DNA ladder. Ethidium-bromide-stained gels were visualized and photographed under 260 nm UV light. (**B**,**C**) Effect of Rlip depletion on DNA fragmentation in MDA-MB-231 and MCF7 cells as measured by TUNEL. Cells were transfected with Rlip-LNA or control antisense (CAS) for 24 hours, as described in the Materials and Methods. After Rlip depletion, the apoptotic intensity was determined by flow cytometric TUNEL assay. (**B**) Logarithmic dot plots show the percentage of TUNEL-positive cells in different groups (US = unstained) as measured by flow cytometry. Viable cells were identified by gating on forward and side scatters. (**C**) The overlapped peaks (logarithmic histogram) demonstrate the effects as a whole and are expressed as the fluorescence intensity of the number of counts of the TUNEL-positive cells obtained from the statistical analysis of the fluorescence height and mean value of the x-axis displayed by the software. The fluorescence level for discrimination between apoptotic and nonapoptotic cells was set using the control without TdT (terminal deoxynucleotidyl transferase). Cells above this fluorescence value in the TdT-positive sample were considered apoptotic. Analysis was performed using the BD CSampler software (BD Biosciences). At least 10,000 cells were analyzed per staining. Data were obtained from three independent experiments.

We previously reported that Rlip depletion/inhibition can inhibit cancer cell growth and induce apoptosis [52,53,54]; therefore, we examined whether breast cancer cell death caused by Rlip-LNA was due to apoptosis. The DNA laddering assay was used to examine apoptosis in CAS or Rlip-LNA treated MCF7 and MDA-MB-231 cells (Figure 3A). Apoptotic DNA fragmentation was evident from agarose gel pictures showing the appearance of DNA laddering in MDA-MB-231 and MCF7 cells treated with the Rlip-LNA, but not the control antisense (Figure 3A). The laddering pattern is a consequence of sequential DNA cleavage by caspase-activated DNAase during the execution phase of apoptosis. These results suggest that the rate of apoptosis by Rlip-LNA was similar in the two cell lines. The apoptotic mechanism of cell death by the Rlip-LNA was further validated by a flow cytometric terminal deoxynucleotidyl transferase dUTP nick end labeling (TUNEL) assay. Flow cytometry analysis indicated that the cellular apoptosis rate increased after treatment with Rlip-LNA (200 ng/mL for 24 h) relative to the vehicle control or the CAS (Figure 3B,C). Both the ER+ and TNBC cell lines showed an increase in the rate of apoptosis with Rlip-LNA treatment. It appears from the bimodal MDA-MB-231 scatterplot and histogram (Figure 3B,C) that these cells had a dimorphic population, possibly because of a difference in cell cycle, heterogeneity in growth rate, or acquired partial cross-resistance towards Rlip-LNA treatment. Together, these results support an apoptotic mechanism for breast cancer cell death by Rlip-LNA treatment.

### 2.5. Role of Rlip in Regulating WNT5A Signaling

**Figure 4:**
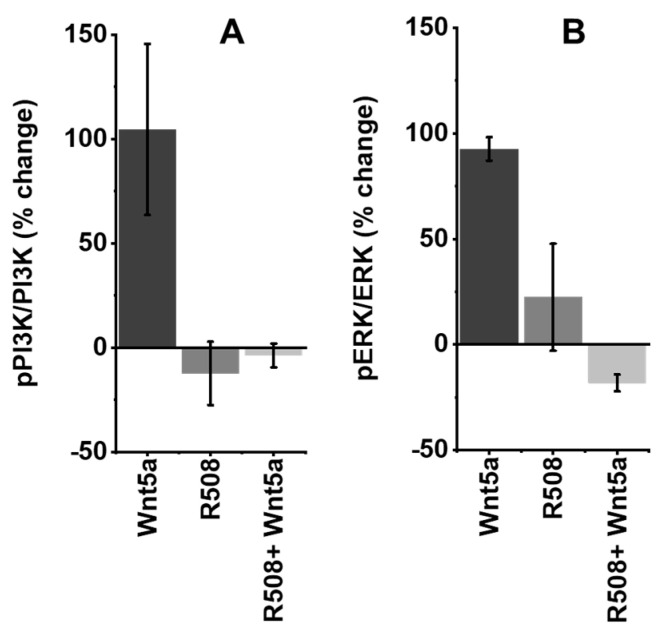
Effect of R508 on WNT5A signaling. ELISA (active motif) assays were used to quantify (**A**) P-PIK3CA and PIK3CA as well as (**B**) P-ERK and ERK 12 hours after treatment, as per the Materials and Methods.

WNT5A is involved in promoting breast cancer, and CDE is known to differentially modulate WNT signaling [36]; thus, blockade of Rlip function should disrupt WNT signaling. MYC is the primary transcription factor targeted by canonical WNT signaling [55], whereas non-canonical WNT signaling can activate PI3K, AKT, and ERK, kinases which are also activated by HER2 signaling. CDE is a RAL/RAC/RHO-regulated process that modulates intracellular signaling cascades by initiating internalization of ligand–receptor complexes in the plasma membrane and by regulating the sorting process that directs the trafficking of endocytic vesicles between intracellular organelles [34,36]. Rlip and its binding partners POB1 and epsin are components of the CDE complex [34,36]. MEFs (mouse embryonic fibroblasts) from Rlip-knockout mice are severely deficient in CDE [34,38]. To investigate a potential role of Rlip in WNT signaling, we depleted Rlip using the R508 Rlip antisense in MCF7 cells, followed by treatment with WNT5A protein for 12 hours. Treatment with WNT5A doubled the ratio of phosphorylated to un-phosphorylated PIK3CA, showing that this non-canonical WNT pathway also operates in MCF7 breast cancer cells (Figure 4A). Rlip depletion by itself had no effect on this ratio, and WNT-mediated PIK3CA phosphorylation was completely abrogated by Rlip depletion (Figure 4A). Similarly, treatment with WNT5A protein activated ERK whereas Rlip depletion by R508 did not, and Rlip depletion abrogated WNT5A-mediated ERK activation (Figure 4B). Supplemental Data S13 shows that apoptotic DNA laddering was minimal or nonexistent at 12 hours after Rlip knockdown. Thus, it is unlikely that the observed decrease in PIK3CA and ERK phosphorylation was due to the death of the cells, although it is possible that early apoptotic signaling events had been initiated by 12 h.

### 2.6. Rlip-LNA Inhibits Endocytosis in Breast Cancer Cells

**Figure 5:**
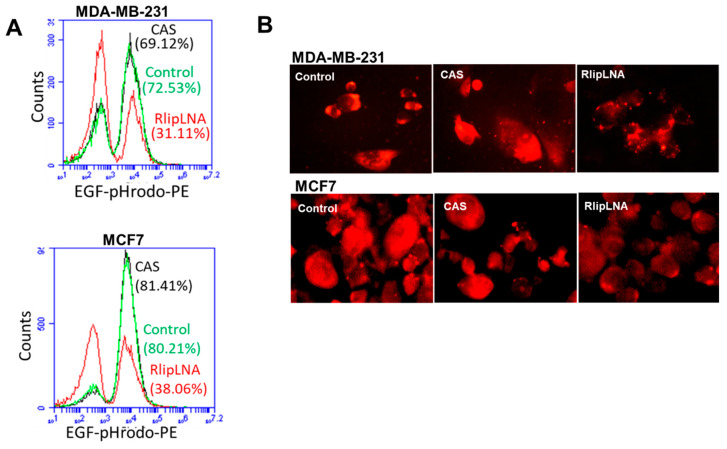
Effect of Rlip depletion on EGF binding and internalization. Cells were transfected with Rlip-LNA or control antisense (CAS), as described in the Materials and Methods. (**A**) Logarithmic histograms expressed as the fluorescence intensity of number of counts of the EGF-pHrodo-positive cells obtained from the statistical analysis of the fluorescence height and mean value of the x-axis displayed by the software. Data were obtained from three independent experiments. The fluorescence level for discrimination between EGF-pHrodo-positive and -negative cells was set using the unstained control. Viable cells were identified by gating on forward and side scatters. At least 10,000 cells were analyzed per staining. (**B**) Representative microscopic images of EGF-pHrodo staining for EGF internalization from three separate experiments for Rlip-LNA transfected, CAS, and vehicle control.

CDE is a key process in vesicular trafficking. It is responsible for the transport of biomolecules, for remodeling the plasma membrane, and for regulating cell-surface signaling [56,57]. We previously demonstrated that the rate of GS-E efflux by Rlip mutants correlates directly with its anti-apoptotic activity and with the rate of endocytosis of EGF and insulin [34]. We recently observed that depletion of Rlip protein also inhibits CDE in melanoma and lung cancer [58,59]. To determine whether LNA-mediated depletion of Rlip is also relevant in inhibition of CDE in breast cancer cells, we investigated the effect of Rlip depletion by antisense on the endocytosis of EGF-rhodamine. Using flow cytometry quantitation (Figure 5A), and fluorescence microscopy (Figure 5B), we found that endocytosis of EGF-rhodamine was markedly reduced in LNA-transfected MCF7 and MDA-MB-231 cells. We performed immunofluorescence staining to confirm the flow cytometry results by fluorescence microscopy. Results demonstrated that the fluorescence was distributed throughout the cytoplasm of control antisense (CAS) or vehicle-treated cells, whereas in cells transfected with Rlip-LNA, the rhodamine signal remained localized near the plasma membrane (Figure 5B) as we showed previously [58]. The inhibitory effects of Rlip-LNA on endocytosis were also similar to those observed previously by depletion of Rlip by the R508 antisense and were consistent with previous studies showing that the endocytic process is interrupted soon after internalization, leaving vesicles stalled along the plasma membrane [38,58]. In conclusion, our results demonstrated that inhibition of EGF endocytosis and induction of apoptosis by Rlip depletion are possible mechanisms of anticancer effects in the human breast cancer cell lines MCF7 and MDA-MB-231.

### 2.7. Anti-Neoplastic Activity of Rlip Targeting in Breast Cancer Xenografts

**Figure 6:**
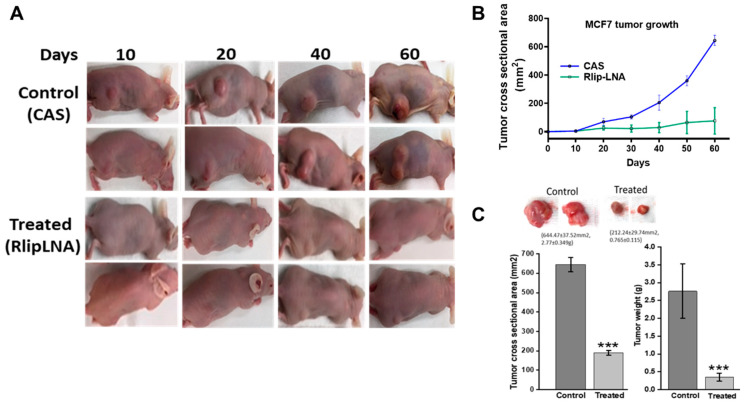
Effect of Rlip-LNA on growth rate of MCF7 tumors. (**A**) Monitoring tumor burden and treatment response. Representative images of the two groups, with two mice from each group (CAS-control and Rlip-LNA-treated) of MCF7-tumor bearing mice. Images are from Day 10 to the end of the study. Tumor weights were determined at the end of the study (Day 60) for each group. Tumor growth was monitored every week, and the tumor sizes were determined using a caliper to measure the tumor cross-sectional area (mm^2^), calculated in two dimensions using the formula A = (D_1_ × D_2_)/2. (**B**) Cross-sectional area of the tumor. Figure shows the growth of tumors in the control and treated groups from Day 10 to the end of the study. (**C**) Histogram shows the tumor cross-sectional areas and tumor weights at the end of the study. Results are reported as mean ± SD. (*n* = 5 each group). *** *p* < 0.001, as compared to the control group of mice, as analyzed by two-tailed Student’s *t*-test.

**Figure 7:**
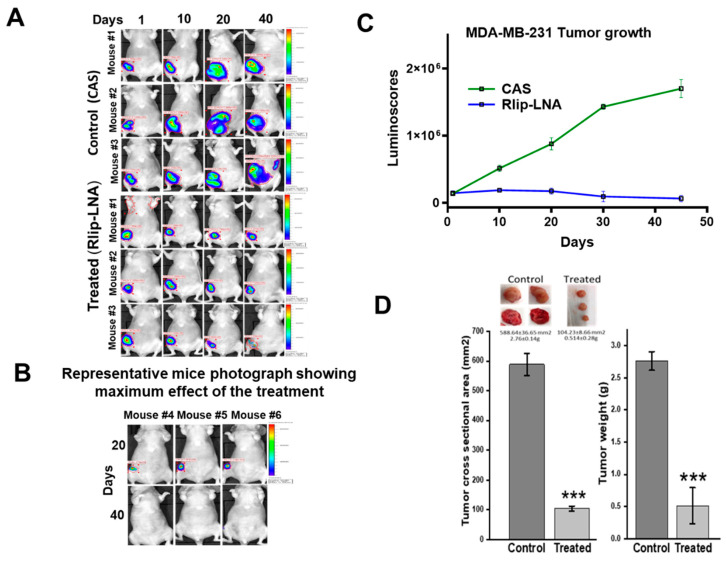
Effect of Rlip-LNA on growth rate of MDA-MB-231luc tumors. (**A**) Tumor burden was monitored via the luminoscore. Cells were injected as described in the Materials and Methods. Five mice per group were used and the experiment was repeated once. Seven drug injections were given. Control mice received the same number of injections of CAS in an equivalent volume (200 μL). Images shown are from three mice from each group from Day 10 to the end of study. As seen, the effect of Rlip-LNA on the luminoscore was very significant on Day 40. (**B**) Panel shows the images of the three Rlip-LNA-treated mice that showed the greatest effect of treatment at Days 20 and 40. Disease was undetectable by the study endpoint. (**C**) Plot shows weekly tumor growth as measured by IVIS imaging, and units are in luminoscores for each group. Rlip-LNA-treated mice showed significant (*** *p* < 0.001) reduction in luminescence at each time point. (**D**) Histogram shows the tumor cross-sectional areas and tumor weights at the end of the study. Results are reported as mean ± SD. *** *p* < 0.001, as compared to the untreated group of mice, as analyzed by two-tailed Student’s *t*-test.

The *in vitro* anticancer activity of Rlip-LNA against breast cancer cells predicted that Rlip depletion should correlate with slower tumor growth and improved survival. To confirm the results of the *in vitro* studies, we conducted *in vivo* studies on the antineoplastic efficacy of Rlip depletion by antisense in xenograft models of breast cancer. The inhibitory effect of Rlip-LNA on tumor growth was tested in athymic nude mice with MCF7 (Figure 6) and MDA-MB-231luc (Figure 7) tumor xenografts. Treatment with 8 mg/kg body weight Rlip-LNA was started one day after tumor inoculation. Administration of Rlip-LNA showed potent antitumor activity in MCF7 and MDA-MB-231 xenografts (Figure 6, Figure 7 and Figure S2) and also resulted in fewer tumor metastases upon gross examination. Treatment significantly (*p* < 0.001) inhibited the MCF7 and MDA-MB-231luc xenograft tumor growth, with 82% and 85% reduction of tumor cross-sectional area and 63% and 62% reduction in tumor weight when compared to antisense controls, respectively (Figure 6C and Figure 7D). In MCF7 xenograft tumors, out of 5 treated, 3 mice showed a 90% tumor regression, and in MDA-MB-231luc tumor-bearing mice, 3 of 10 showed complete regression and 2 achieved 90% regression as measured by IVIS imaging.

To validate our findings of Rlip-LNA activity, we also tested the efficacy of polyclonal antibodies against the aa^171-185^ Rlip peptide using the respective pre-immune antibodies as controls. While MCF7 tumors in control mice enlarged, tumors in mice treated with anti-Rlip antibodies decreased in size for the duration of the study (Figure S1B,C). It appears that the anti-tumor activity of Rlip antibody was equivalent to, if not better than, Rlip-LNA in *in vivo* models; thus, the LNA may be optimally used as a research tool, while the antibody may hold more clinical promise due to its superior pharmacology. Both the Rlip-LNA and the anti-Rlip antibody treatments were well tolerated by the mice as assessed by weight loss (Figure S3A–C).

### 2.8. Targeting Rlip Regulates Cancer Signaling in MCF7 and MDA-MB-231 Xenografts

**Figure 8:**
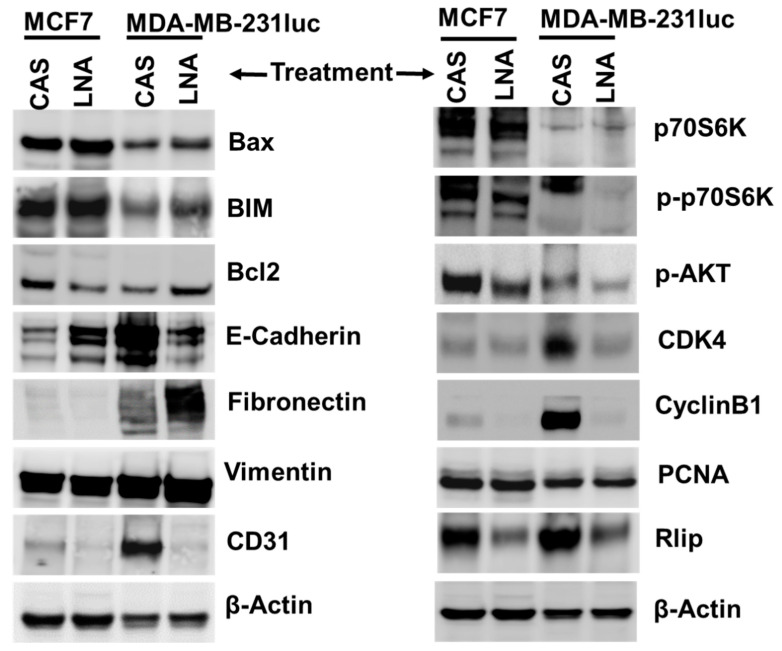
Effect of Rlip depletion with Rlip-LNA on the levels of cell survival, proliferation, apoptosis, and differentiation marker proteins in MCF7 and MDA-MB-231luc breast cancer xenograft tumors. Western blots from lysates of tumors collected at study endpoints showing apoptosis, cell survival, proliferation, and differentiation marker proteins in MCF7 and MDA-MB-231luc tumor tissue lysates after Rlip-LNA treatment. Representative Western blots are shown in the figure. β-actin was used as a loading control.

Western blot analysis indicated that several cancer hallmark pathways were suppressed following Rlip-LNA treatment. Expression of anti-apoptotic BCL2 was reduced in MCF7, and the expression of pro-apoptotic BIM and Bax was increased in both MCF7 and MDA-MB-231luc xenograft tumors (Figure 8 and Supplemental Data S16,S17). Microvessel density and angiogenesis marker CD31 was also decreased in both MCF7 and MDA-MB-231, compared to controls. Rlip depletion increased epithelial marker E-cadherin in MCF7 tumors, but it was decreased in MDA-MB-231. In contrast, mesenchymal marker fibronectin was diminished in MCF7 but increased in MDA-MB-231 tumors, as observed by Western blot analyses (Figure 8 and Supplemental Data S16–S18). This difference in the expression of apoptotic and epithelial/mesenchymal proteins between the two cell line xenografts might have been due to the differential activity of Rlip protein between the two cell lines. Additionally, results for both xenograft models indicated that Rlip-LNA treatment inhibited proliferation and cell cycle progression, as evidenced by decreased CDK4 and CyclinB1 protein levels, and inhibited the activation of the Akt/P70S6 kinases as seen by a reduction in phosphorylation. Finally, Rlip was depleted by Rlip-LNA in both xenograft models. (Figure 8 and Supplemental Data S16,S19,S20). Tumor origin of excised tissues was verified by hematoxylin and eosin (H&E) staining (Supplemental Data S14). IHC stains of MCF7 tumor tissue sections showed a remarkable disappearance of the proliferative marker Ki67 and the angiogenesis marker CD31 (Figure S1D). E-cadherin, a marker of differentiation that signifies reversal of epithelial–mesenchymal transition, also increased remarkably with R508 (Figure S1D). These results further corroborated the finding that the targeting of Rlip results in anticancer activity against breast cancer.

## 3. Discussion

Previous studies have established that Rlip facilitates the efflux of GSH conjugates of toxic cellular metabolites and drugs from cells to modulate cancer cell survival. An unprecedented suppression of malignancy by hetero- or homologous loss of Rlip in p53-null mice, along with p53 abnormality in the majority of breast cancers, suggests an existential importance of Rlip in breast cancer. Here, we showed that depletion/inhibition of Rlip in breast cancer using a nucleotide 508-529-targeted locked nucleic acid (LNA), a third-generation antisense technology with improved pharmacological properties, led to increased apoptosis, in vitro survival/growth inhibition, altered signaling protein levels, and inhibition of endocytosis. Further, Rlip inhibition prevented the growth of both ER+ and TNBC cell line xenografts, even resulting in three complete responses in MDA-MB-231-grafted mice by the study endpoint. The equivalent in vivo efficacy observed with two independent targeting methods, including Rlip-LNA and Rlip antibodies in the present studies, as well as anti-Rlip antibodies, R508 antisense, and Rlip-siRNA in prior studies, essentially rules out any significant off-target therapeutic effects. Our study showed that in vitro Rlip deficiency inhibits EGF endocytosis and downstream signaling from WNT5A to ERK and PIK3CA, which are key proteins involved in the oncogenesis and natural history of breast cancer. This was corroborated by in vivo findings that markers of proliferation, angiogenesis, apoptosis, and cell cycle progression were reduced in MCF7 and MDA-MB-231 tumor lysates following Rlip depletion, and the Western blot results indicating changes to proliferation, angiogenesis, and adhesion in MCF7 were supported by IHC studies. The epithelial and mesenchymal markers E-cadherin and fibronectin were affected in an opposite manner by Rlip inhibition in MCF7 and MDA-MB-231luc tumors. The reason for the difference is not presently clear, although it may have been due to differences in ER+ and TNBC biology. It is also worth noting that MDA-MB-231 cells showed a bimodal response to Rlip-LNA by TUNEL assay, indicating that the cell line exists as a dimorphic cell population with respect to Rlip function. Because Rlip levels are cell-cycle-dependent in shutting down endocytosis at cell division, this finding might have been due to a difference in the population of cells undergoing mitosis.

These findings add breast cancer to the list of cancers susceptible to Rlip deficiency, joining melanoma, lung cancer, prostate cancer, colon cancer, renal cell carcinoma, pancreatic cancer, and neuroblastoma [27,34,37,38,39,40,41,58,60]. In addition to this remarkably wide spectrum of activity, an existential role of Rlip in cancer progression is supported by the failure of Rlip-knockout mice to develop cancer upon exposure to the powerful carcinogens benzo[a]pyrene and dimethylbenzanthracene, which cause cancer in 100% of wild-type mice [34]. Additionally, the failure of syngeneic B16 melanoma cells to implant in Rlip-knockout mice due to impaired angiogenesis indicates that carcinogenesis requires the presence of Rlip in the cells of the stroma as well as in the cancer parenchyma [61]. Cancer-susceptible p53-null mice develop age-dependent epigenomic and transcriptomic abnormalities that result in increased levels of oncogenes and reduced levels of tumor suppressors [42]. The abnormal CpG island methylation and altered expression of many oncogenes and tumor suppressors are reversed when Rlip is reduced through administration of Rlip antisense oligonucleotides or through crossbreeding with Rlip-null mice [42]. Lost or altered p53 function also occurs in a large fraction of human breast cancers because of the high frequency of p53 mutations or deletions [43]. Thus, we conjectured that the mechanism of action of breast cancer regression upon Rlip depletion involved broad-spectrum effects on oncogenes and tumor suppressors that resembled those found in Rlip-null mice or p53-null mice with Rlip deficiency. By querying METABRIC data using cBioportal and IPA (Ingenuity Pathway Analysis, Qiagen) network analyses, we found that the majority of genes most frequently altered in human breast cancers were also transcriptionally altered in p53^−/−^ mice and that the direction of change in z-scores (cutoff 1.6) caused by Rlip depletion in these mice resembled those previously reported in Rlip+/− mice (Supplemental Data S11A–C) [42]. The experiments presented in this paper confirmed that Rlip depletion in human breast cancer cell lines does indeed affect several of the breast cancer signaling pathways that are abnormal in p53^−/−^ mice and reverted by Rlip depletion [42].

Interestingly, these analyses also revealed that the mutation and CNA frequencies of breast cancer genes were significantly different when comparing the upper vs. lower quartile of Rlip expression (Supplemental Data S1A–E, S2–S10). TCGA analyses showed that *MYC*, *PIK3CA*, and *CDKN2A/B* are the most frequently amplified, mutated, and deleted genes, respectively, in breast cancer. The importance of these proteins in breast cancer is well established experimentally. The fact that these pathways are regulated by Rlip, as observed in the present experimental studies, taken together with the fact that there are distinct differences in the frequency of mutations, amplifications, and deletions of these and other important genes in breast cancer with respect to Rlip expression, indicates that the interaction of Rlip with these proteins plays a fundamental role in the biological behavior of breast cancer. Combined analyses of the TCGA, GEO, and EGA databases supported this assertion because Rlip expression status among patients was clearly associated with better or worse overall survival (OS) and relapse free survival [62], and Rlip expression was further associated with alterations of many clinically validated biomarkers used to classify breast cancers according to biological behavior, prognosis, and predicted efficacy of nearly all presently used anticancer therapies.

Because our genomic studies showed clear interactions of Rlip with *MYC* and *PIK3CA*, the different frequencies of CNAs and mutations in these genes between patients with low or high Rlip expression could result in context-specific effects of Rlip on survival. Additionally, previously observed functional interactions between Rlip and p53 suggest the possibility that Rlip, by an unknown mechanism, is associated with genetic instability, and Rlip itself could be a determinant of the frequencies of certain types of genetic alterations. We previously hypothesized that a haploinsufficiency mechanism involving altered complexing between HSF1, p51, and Rlip may contribute to carcinogenesis [42], and the role of p53 in defending the integrity of the genome [20] could potentially underlie this observation. Furthermore, a role of Rlip in mediating drug resistance [7,9,10,38,60] could influence survival differentially between patients treated with estrogen deprivation versus chemotherapy.

Together, these prior studies established that Rlip is a multifunctional stress-responsive and anti-apoptotic protein that functions as a nexus between the biochemical pathways that utilize GSH to remove electrophilic endobiotic and xenobiotic toxins from cells, and the cancer-promoting signaling mechanisms that lie downstream of the ligand–receptor signaling networks that are regulated by CDE [34]. The survival of homozygous Rlip-knockout mice without embryonic lethality indicates that its activities are relatively dispensable in normal unstressed cells [14], an essential characteristic of an intrinsically cancer-specific target. This predicts a lack of significant systemic toxicity upon pharmacologically induced depletion of Rlip in the whole animal. This notion is supported by several animal studies showing no substantial toxicity when R508 is used to cause systemic depletion of Rlip without any cancer-specific targeting [27,37,39]. Perhaps more importantly, the anti-metabolic-syndrome actions of Rlip depletion may provide additional beneficial effects in patients with metabolic syndrome, the prevalence of which is between 35% and 40% in the U.S. population [50,63,64]. Metformin, a drug used to reduce blood sugar in diabetes patients, has been shown to protect against breast cancer and also to inhibit Rlip-transport activity [64,65]. Interestingly, blood sugar in Rlip^-/-^ mice is not reduced by metformin [64].

The wide-spectrum and excellent antineoplastic activities of R508 and anti-Rlip antibodies in animal models indicate that Rlip-targeted therapeutics should be highly effective in the treatment of human cancers, because Rlip may be of fundamental importance for the existence of cancer cells. The near-complete resistance of Rlip-knockout mice to chemical carcinogenesis and the failure of mouse melanoma implantation in Rlip-knockout mice due to impaired angiogenesis also indicate a broader importance for Rlip in cancer [61]. The facts that Rlip is not lost in 99.9% of all cancers in TCGA (n = 19,283 cases) and that amplifications involving Rlip are 13 times as frequent as deletions further strengthen this assertion of existential importance.

Rlip regulates the metabolism of electrophilic and oxidative xenobiotics to mercapturic acids, a pathway that is essentially never lost in cancer cells. Enzymes constituting this pathway have been repeatedly identified as being increased in cancers. Rlip represents the last biochemical step of this pathway in cells, after which the remainder of the transformation to mercapturic acid occurs primarily through the actions of renal enzymes, γ-glutamyl-transpeptidase, dipeptidases, and N-acetyl transferases [66,67]. Loss of Rlip should therefore cause an accumulation of all precursor metabolic intermediates, many of which are pro-apoptotic. Product and feedback inhibition of key upstream mercapturic-acid-pathway enzymes by glutathione conjugates [68,69] would exacerbate this condition. This has been demonstrated in Rlip-knockout mice, which have high levels of GS-E, upstream lipid-hydroperoxides of polyunsaturated fatty acids and their degradation products, and potent pro-apoptotic alkenals, as well as inhibition of activity of multiple glutathione utilization enzymes [14,70]. The rate-limiting role of Rlip in endocytosis also contributes to the unique position of Rlip, linking it both to signaling pathways important for the formation and growth of cancer and to the potent anticancer effects of targeting Rlip [34].

## 4. Materials and Methods

### 4.1. Reagents

Anti-Rlip, anti-Ki67, anti-CD31, anti-E-cadherin, anti-Bax, anti-Bim, anti-Bcl2, anti-Fibronectin, anti-vimentin, anti-p70s6k, anti-AKT, anti-CDK4, anti-cylinB1, anti-PCNA, anti-beta-actin, and negative control IgG were purchased from Santa Cruz Biotech (Santa Cruz, CA, USA). FACE-ERK1/2 and FACE-PI3K kits were purchased from Active Motif (Carlsbad, CA, USA). WNT5A was obtained from R&D Systems (Minneapolis, MN, USA). NHS-rhodamine, pHrodo™ Red Epidermal Growth Factor (EGF) Conjugate, and Lipofectamine 2000 were purchased from Thermo Fisher (Waltham, MA, USA). Rlip-LNA and LNA control antisense (CAS) were purchased from Exiqon (Woburn, MA, USA). Phosphorothioate Rlip-antisense (R508) and phosphorothioate control antisense (CAS) were purchased from Biosynthesis Inc. (Lewisville, TX, USA). Sources of other reagents were the same as previously described [71,72,73]. All other reagents were of analytical grade.

### 4.2. Cell Lines and Cultures

Human breast adenocarcinoma cell lines MDA-MB-231, MDA-MB-231-luc-D3H2LN (luciferase expressing cell line), and MCF7 were cultured in DMEM (American Type Culture Collection), supplemented with 10% fetal bovine serum (ATCC), 100 U/mL penicillin, and 100 µg/mL streptomycin (Invitrogen Life technologies, Carlsbad, CA, USA) in a 5% CO_2_ incubator at 37 °C [9]. The Integrative Genomics Core (Texas Tech University Health Sciences Center, Lubbock, TX, USA) analyzed 15 different human short tandem repeats (STRs) to authenticate the cell lines and test for interspecies contamination. Cells were also tested for Mycoplasma once every 3 months.

### 4.3. Mouse Studies

Female nude mice aged 5-6 weeks old were obtained from Jackson Laboratories (Bar Harbor, ME, USA). Mice were housed and maintained in the animal care units at Texas Tech University Health Sciences Center (TTUHSC), Lubbock, TX. Mice were given ad libitum access to water and chow (Harlan Teklad, Madison, WI, USA). The mice were housed at an ambient temperature of 22 °C ± 2 °C, hygrometry of 45% ± 10%, with 12/12h light/dark cycles, and were acclimatized for 1 week before starting the experiments.

### 4.4. Ethics Statement

This study was carried out in strict accordance with the recommendations of U.S. National Institutes of Health Guide for the Care and Use of Laboratory Animals. The Institutional Animal Care and Use Committee (IACUC approval #18015) approved the protocol. All procedures were performed under anesthesia and all efforts were made to minimize pain and suffering of the animals.

### 4.5. R508 Antisense and Locked Nucleic Acid (LNA) Oligonucleotides of Rlip

The oligonucleotide sequence (R508) corresponding to nucleotides 508–529 (5′GGCTCCTGAATTGGCTTTTTC 3′) of the N-terminal protein coding region was found to have the least homology with other known sequences. The 3D model of the Rlip-antisense was predicted using RNAComposer online server. The RNA model was then translated into an ssDNA model by replacing base U with T and converting the RNA backbone into a DNA backbone using the VMD psfgen Plugin [74]. The initial model was further refined by performing 4ns molecular dynamics simulation using NAMD [74]. The control scrambled antisense (CAS) was among several scrambled control sequences generated using GenScript software, and was chosen based on a lack of effects on known activities of Rlip in cultured cells [39]. The fully phosphorothioated antisense (R508) and the phosphorothioate CAS were synthesized and purified using HPLC by Biosynthesis Inc. (Lewisville, TX), who provided 50 mg lyophilized vials of antisense that were purified by HPLC to >90% purity, and authenticity was validated by MALDI-TOF and elemental composition analysis. The Rlip-depleting activity of R508 and Rlip-LNA and the lack of this activity with CAS were confirmed in lung, melanoma, and breast cancer cell lines. The R508 and Rlip-LNA sequence bears homology to HTRA2 (serotonin receptor, 76%) and CAS to FBXL (F-box-like 16 gene, 61%), but the expression of neither gene was altered significantly at 24 h after treatment of MCF7 cells with the respective antisense/scramble molecules (data not presented). The antisense molecules were dissolved in PBS to prepare stock solutions of 10 mg/mL and dilutions were used for in vitro assays and for treatment of nude mice bearing tumor xenografts.

### 4.6. Cell Proliferation and Cytotoxicity Assay

Cultured adherent MDA-MB-231 or MCF7 cells were trypsinized and pelleted by centrifugation at 500 g for 5 min at 4 °C and washed twice by suspension in complete DMEM; cells were counted using a Z1 COULTER COUNTER cell and particle counter (Beckman Coulter Inc., Brea, CA, USA). For cell viability assays, cells were seeded at 1 × 10^5^ cells/mL in DMEM and 100 µL/well in 96 well plates and allowed to recover for 16–18 h. The next day, one set of cells was transfected with 0.1 µg/well CAS, R508, or Rlip-LNA using Lipofectamine 3000, and another set of cells was treated with 20 µg/well control IgG, Rlip-polyclonal antibody, or Rlip-monoclonal antibody. After incubation at 37 °C for 48 h, cell proliferation was assayed as described previously using MTT [58]. For colony-forming assays, 1 × 10^5^ cells / 500 µL were incubated in 10 µg/mL R508 or scrambled-antisense for 24 h, and then aliquots of 50 or 100 µL were added to 60 mm Petri dishes containing 4 mL culture medium. After 10 days, adherent colonies were stained with methylene blue for 30 min and counted.

### 4.7. Effect of Rlip Depletion on EGF Binding and Internalization Immunofluorescence

The effect of Rlip knockdown/depletion by Rlip-LNA and the respective scrambled control antisense (CAS) on EGF endocytosis was studied in the MCF7 and MDA-MB-231 cell lines by immunofluorescence and flow cytometry analysis. The cells were transfected by reverse transfection. Briefly, Rlip-LNA, CAS and Lipofectamine complexes were prepared as follows: Rlip-LNA or CAS (10 µg/mL final concentration) was diluted in 500 μL serum-free Opti-MEM Medium and added to culture wells directly, and 4 µL of Lipofectamine 3000 reagent was added to each well containing antisense RNA complex. The complex was mixed gently and incubated at room temperature for 30 min. Cells were suspended in complete growth medium without antibiotic. After 30 min, 500 µL of cell suspension containing 4 × 10^4^ cells was added to each well, having antisense oligo and Lipofectamine complex. Contents were mixed gently. Cells were incubated in a 5% CO_2_ incubator at 37 °C. After 24 h, cells were kept on ice for 20 min then washed with cold live-cell-imaging solution (LCIS) containing 2 mM glucose and 1% BSA, and incubated with 2 µg/mL EGF-pHrodo (Invitrogen) according to the manufacturer’s instructions. Cells were then incubated at 37 °C in a humidified chamber for 30 min followed by washing with LCIS. DAPI solution (0.02 µg/mL) applied for 5 min was used to stain nuclei. DAPI solution was removed and citrate buffer was added to each well. Slides were analyzed using a fluorescence microscope (Olympus America, Melville, NY, USA). Photographs taken at identical exposure settings at 200× magnification are presented. The effect of Rlip knockdown on endocytosis was further checked by flow cytometry.

### 4.8. Flow Cytometric Analysis of EGF Endocytosis

For flow cytometry analysis, cells were grown on 60 mm tissue culture dishes. Rlip knockdown using Rlip-LNA or control antisense was performed as described above. After 24 h, cells were trypsinized, washed with cold LCIS buffer, and counted. Cell samples (1 × 10^6^ cells) were incubated on ice for 20 min, after which samples were centrifuged at 200× g for 5 min followed by washing with LCIS at room temperature. After washing cells were incubated with 2 µg/mL EGF-pHrodo complex in LCIS at 37 °C for 45 min, washed with cold staining buffer, incubated for 10 min at 37 °C, and analyzed with the BD Accuri C6 Flow Cytometer (BD Biosciences, San Jose, CA, USA). The fluorescence level for discrimination between EGF-pHrodo-positive and -negative cells was set using the unstained control. Viable cells were identified by gating on forward and side scatters. At least 10,000 cells were analyzed per staining. Data are shown as a logarithmic histogram and expressed as fluorescence intensity of number of counts of the EGF-pHrodo-positive cells obtained from the statistical analysis of the fluorescence height and mean value of the x-axis displayed by the software. Data were obtained from three independent experiments.

### 4.9. TUNEL Assay

A terminal deoxynucleotidyl transferase dUTP nick end labeling (TUNEL) assay was utilized to assess and validate apoptotic cell death. The assay for FACS analysis was performed using an APO-BrdU TUNEL assay kit (Invitrogen, Thermo Fisher Scientific). The cells were transfected with Rlip-LNA for 24 hours by reverse transfection as above. After transfection, cells were processed for staining following the manufacturer’s protocol. In brief, cells were washed with PBS and trypsinized. Cells (1–2 × 10^6^) were suspended in 0.5 mL PBS and added to freshly prepared 1% (w/v) buffered paraformaldehyde, and placed on ice. After 15 min, cells were washed twice with PBS, and ice-cold 70% (v/v) ethanol was added to the cells before placing on ice for 30 min. Cells were washed twice with wash buffer (provided with kit), resuspended in 50 µL of DNA-labeling solution, and incubated for 60 min at 37 °C in a water bath. Cells were rinsed two times with rinse buffer (provided with kit). Cell pellets were resuspended in 100 µL of antibody solution and further incubated for 30 min at room temperature in the dark. After incubation, cells were analyzed with the BD Accuri C6 Flow Cytometer (BD Biosciences, San Jose, CA, USA). The fluorescence level for discrimination between apoptotic and nonapoptotic cells was set using the control without TdT (terminal deoxynucleotidyl transferase). Cells above this fluorescence value in the TdT-positive sample were considered apoptotic. The percentages of cells undergoing apoptosis were assessed. Analysis was performed using the BD CSampler software (BD Biosciences). Viable cells were identified by gating on forward and side scatters. At least 10,000 cells were analyzed per staining. Data are shown as logarithmic dot plots and histograms, and expressed as mean fluorescence intensity and number of counts of the TUNEL-positive cells, obtained from the statistical analysis of the fluorescence height and mean value of the x-axis displayed by the software. Data were obtained from three independent experiments.

### 4.10. Determination of Apoptosis by DNA Laddering

Aliquots of 3 × 10^6^ cells/6 mL medium were grown in 6 well plates and next day transfected with Rlip-LNA or CAS using Lipofectamine 3000. After 48 h incubation at 37 °C, cells were collected, washed with PBS, and subjected to DNA extraction according to the instructions in the Apoptotic DNA Ladder Kit (Invitrogen). Agarose gel electrophoresis was performed in 2% agarose with a 1 kB DNA ladder. Ethidium-bromide-stained gels were visualized and photographed under 260 nm UV light [75].

### 4.11. Subcellular Localization of Rlip by Electron Microscopy

Immunogold EM was performed in the City of Hope Core facility with standardized methods as follows: MCF7 cells were cultured in RPMI-1640 medium containing 10% FBS and 1% P/S. Cultured cells were pelleted and cryo-fixed in a Leica EM PACT2 high pressure freezer (~2000 bars). Cryo-fixed specimens were freeze-substituted in anhydrous acetone containing 0.5% glutaraldehyde and 0.1% uranyl acetate using the AFS2, a Leica automated freeze-substitution system (temperature progression −90 °C × 48 h, increased from −90 °C to −50 °C over 8 h, and then incubated at −50 °C for 16 h). After rinsing with acetone three times for 20 min each at −50 °C, the specimen was infiltrated with 50% Lowicryl HM20 for 2 h, 67% Lowicryl HM20 for 2 h, and 100% Lowicryl HM20 overnight with two exchanges. The specimen was then polymerized in Lowicryl HM20 for 24 h at −35 °C under a UV lamp. Polymerization was continued for an additional 48 h at room temperature. Ultra-thin sections (~70 nm thick) were cut using a Leica Ultra cut UCT ultra-microtome with a diamond knife on 200 mesh nickel EM grids. Grids were stained with 2% uranyl acetate in 70% ethanol for 1 min, followed by Reynold’s lead citrate staining for 1 min. Post-embedding immunolabeling was carried out with a 1:100 dilution of Rlip mouse monoclonal primary antibody. The antigens were detected with 15 nm colloidal-gold-conjugated secondary antibody. The grids were stained with uranyl acetate and lead citrate as described above. Electron microscopy was done on a FEI Tecnai 12 transmission electron microscope equipped with a CCD camera.

### 4.12. Tumor Xenografts, Treatments, and Imaging

Hsd:athymic nu/nu mice were obtained from Jackson Laboratories (Bar Harbor, ME, USA). All animal experiments were carried out in accordance with a protocol approved by the Institutional Animal Care and Use Committee (IACUC). A total of 1 × 10^6^ MDA-MB-231-luc-D3H2LN cells were injected orthotopically into an abdominal mammary fat pad, in 50 µL of serum- and antibiotic-free DMEM. Five mice/group were used for each experiment, and the experiment was conducted twice. After randomization to treatment groups, mice were given 8 mg/kg body weight of Rlip-LNA or CAS weekly by intraperitoneal injection, in ~200 µL of PBS. Primary tumor growth as well as metastases was determined weekly by in vivo bioluminescence imaging using an IVIS LUMINA XR in vivo imaging system (CALIPER LIFE SCIENCES). Mice were gas anesthetized with 1%–3% isoflurane, and substrate luciferin was injected into the intraperitoneal cavity at a dose of 150 mg/kg body weight approximately 10–15 minutes before imaging. Abdominal, ventral, and dorsal images were collected for 1 minute and images were quantified using Living Image software. On Day 40 from the start of treatment, mice were euthanized. An image of each mouse was acquired before sacrifice and tumors were isolated and weighed. Tumors were measured using a caliper and the tumor cross-sectional area (mm^2^) was calculated in two dimensions using the formula (D_1_ × D_2_)/2, where D_1_ and D_2_ represent the tumor length and the tumor width, respectively.

The MCF7 cell line was used to create a xenograft model for the evaluation of Rlip-LNA in ER-positive breast cancer tumors. For the MCF7 xenograft, 5–6 week old nude mice (5 mice/group, total 10) were used for the experiment. A pellet containing 0.72 mg of 17β-estradiol (90 day release, Innovative Research of America, Sarasota, FL, USA) was implanted s.c. into the shoulder area of mice 5 days before tumor cell injection. Subsequently, 1 × 10^6^ MCF7 cells were injected subcutaneously into one flank in 0.1 mL PBS and mice were randomized for the treatment into two groups. After randomization, one group of mice was treated once weekly with 8 mg/kg body weight of Rlip-LNA, IP, in a 0.2 mL volume. Control mice were injected with 0.2 mL of control antisense (CAS). The mice were examined daily for signs of distress and tumor growth. Tumor growth was monitored every week using a caliper and the tumor volumes (cross-sectional area mm^2^) were calculated in two dimensions using the formula (D_1_ × D_2_)/2, where D_1_ and D_2_ represent the tumor length and the tumor width, respectively.

For the antibody validation of Rlip-inhibition/depletion-mediated MCF7 xenograft regression, 12 female Hsd:athymic nu/nu mice were divided into two groups of six animals treated with pre-immune serum or Rlip-antibody. All mice were subcutaneously implanted with a 0.72 mg 90 day release 17-β-estradiol pellet (Innovative Research of America, Sarasota, FL, USA) on the lateral side of the neck. All 12 animals were injected subcutaneously with 1 × 10^6^ MCF7 breast cancer cells suspended in 100 μL PBS. Animals were examined daily for signs of tumor growth. Treatment was administered when the cross-sectional area exceeded 42 mm^2^ (~Day 31 after implantation). Treatment consisted of 200 μg of Rlip-antibodies in 100 μL PBS given intraperitoneally. Control mice were treated with 200 μg/100 μL of pre-immune serum. Tumors were measured in two dimensions using calipers.

### 4.13. Assessment of Angiogenesis, Proliferation, and Apoptotic Signaling

Tissue lysates were prepared from tumors and a full Western blot panel was performed for all mice that had detectable tumors at the endpoint, resulting in n ≥ 3 for all treatment cohorts. Total tissue lysates were loaded on 4–12% Bis-Tris gels (45 µg protein/lane), with 1 × MES gel running buffer or on Tris-acetate gels with acetate buffer. Proteins were transferred to nitrocellulose membrane, and blocking was done in 1× Pierce Clear Milk (Thermo) with 0.1%TWEEN 20 for 1 h at room temperature. Membranes were probed with rabbit mono/polyclonal primary antibodies for BCL2, Cyclin B1, CDK4, p70S6K, phospho-p70S6K (Invitrogen), p-AKT, BIM, and Vimentin (Cell Signaling Technology), and mouse monoclonal antibodies for Bax, E-Cadherin, Fibronectin, Rlip, PCNA, β-actin (Santa Cruz Biotechnology), and CD31 (Abcam). All were diluted to 1:1000 in 1× Clear Milk + 0.1%TWEEN 20 except for p-AKT, which was diluted in 5% BSA + 0.1% TWEEN 20. All were incubated overnight at 4 °C using gentle shaking. Membranes were washed five times (5 min each at room temperature) with Tris-buffered saline-TWEEN 20 (TBST; 20 mM Tris-HCl (pH 7.6), 137 mM NaCl, and 0.2% (*v*/*v*) TWEEN 20) and incubated with horseradish-peroxidase-coupled anti-IgG (secondary antibody, dilution 1:2000) for 1 h at room temperature in 1× Clear Milk + 0.1% TWEEN 20. For visualization of the bands, enhanced chemiluminescence (Super-Signal West Pico Chemiluminescent Substrate; Thermo Scientific, Rockford, IL, USA) was used following the manufacturer’s instructions. For the loading control, at the end of the experiments, nitrocellulose membranes were stripped with Restore Western Blot Stripping Buffer (Thermo Scientific) and re-probed with anti-beta-actin antibody (1:1000 dilution, Santa Cruz Biotechnology). Bands were visualized using an ImageQuant LAS4000 (GE Healthcare Life Sciences, PA, USA).

ELISA analysis of PI3K and ERK phosphorylation was carried out after 12 h incubation using commercial FACE-ERK1/2 and FACE-PI3K kits according to the manufacturer’s protocol (Active Motif, Carlsbad, CA, USA).

### 4.14. Statistical Analysis

All analyses were performed using Prism 6.0 for Windows (GraphPad, SanDiego, CA, USA). Results are reported as mean ± SD. The results were analyzed by two-tailed Student’s *t*-test. A one-way analysis of variance (ANOVA) with post-hoc Tukey’s test was applied when comparing three or more groups. A *p* value of < 0.05 was considered statistically significant. The *p* values presented for genome analyses were from TCGA and KMPlotter, respectively.

## 5. Conclusions

Drug agents for targeting Rlip have not been fully developed. The ability of Rlip to transport a wide variety of antineoplastic agents renders it a multidrug transporter. Other multidrug transporters have not proven to be “druggable”, primarily because of toxicity. This may not be an issue for Rlip, but it remains to be determined. However, the new generation of antisense molecules could be considered. Though liposomal or other nanoparticle formulations were not required in mice, these may be required in humans. In summary, development of Rlip-targeted therapies for breast cancer appears to be justified because of the demonstrated sensitivity of breast cancer models to the strategy, the lack of significant toxicity in laboratory studies in mice, and a reasonably well-defined mechanism of action that involves multiple key cancer-related signaling pathways in breast cancer. Based on our studies, selection of patients on the basis of Rlip expression may not be required, but the presence of genetic alterations in related signaling pathways may be useful as predictive biomarkers in the selection of patients for Rlip-targeting clinical trials.

## Figures and Tables

**Table 1:** Correlation between Rlip expression level and alterations in key signaling pathway genes in breast cancer.

**Amplified Genes**	**Locus**	**Rlip-Unaltered**	**Rlip-High**	**Rlip-Low**	**Function**
*MYC*	8q24.21	25.49	53.42	25.69	proto-oncogene
*RAD21*	8q24.11	24.85	50.68	25.23	sister chromatid cohesion in mitotic cells
*EXT1*	8q24.11	24.39	48.63	24.31	chain elongation step of heparan sulfate
*NDRG1*	8q24.22	23.15	48.63	22.94	stress responses, hormone responses, cell growth, and differentiation
*RSPO2*	8q23.1	23.15	43.15	23.85	signaling receptor binding and G protein-coupled receptor binding
**Deleted Genes**	**Locus**	**Rlip-Unaltered**	**Rlip-High**	**Rlip-Low**	**Function**
*CDKN2A*	9p21.3	2.44	2.05	4.59	cell cycle G1 control
*CDKN2B*	9p21.3	2.44	2.05	4.59	cell cycle G1 progression
*MAP2K4*	17p12	2.12	0.68	2.75	transferase activity and protein tyrosine kinase activity
*PTEN*	10q23.31	2.02	2.74	1.83	multi-functional tumor suppressor
*MTAP*	9p21.3	2.3	2.05	4.59	phosphorylase activity and S-methyl-5-thioadenosine phosphorylase activity
**Mutated Genes**	**Locus**	**Rlip-Unaltered**	**Rlip-High**	**Rlip-Low**	**Function**
*PIK3CA*	3q26.32	42.52	16.55	45.07	transferring phosphorus-containing groups and protein serine/threonine kinase activity
*TP53*	17p13.1	34.66	48.97	45.07	DNA-binding transcription factor activity and protein heterodimerization activity
*MUC16*	19p13.2	16.81	15.86	22.54	cell surface glycosylation
*AHNAK2*	14q32.33	16.71	11.72	19.72	calcium signaling by associating with calcium channel proteins
*SYNE1*	6q25.2	12.38	13.10	12.21	nucleotide binding

The Molecular Taxonomy of Breast Cancer International Consortium (METABRIC) study database was analyzed using MSK cBioportal.org. Of the 2509 total cases, mutation data were available in 2509 cases, from which 12,104 mutations were present in 173 genes. Copy number alteration (CNA) data were available in 2173 cases, from which 1,336,083 CNAs were present in 32,949 genes. The cBioportal database was queried separately for alterations in each gene of interest with respect to CNA, mutation, and mRNA expression by Affymetrix microarray Affy id 202844_s_at. Frequencies are shown as percentages. Data were downloaded for the genes of interest and cases were sorted for each gene based on a Z-score >2 cutoff processed by cBiopotal. *PIK3CA* and *TP53* alterations were overwhelmingly mutations, whereas *MYC* alterations were all amplifications. Rlip CNV alterations were infrequent overall, and there were only two cases in which deletions were found. Data were sorted to identify cases with either mutations, amplifications, or deletions, and these were then sorted by RLIP status. The top five most frequently altered genes with respect to the Rlip-unaltered group are listed.

**Table 2:** Survival in Breast Cancer According to Rlip Expression.

Prognostic Subsets	Relapse-Free Survival (Months)				Overall Survival (Months)			
	Patient Number	Rlip-Low	Rlip-High	-logp	Patient Number	Rlip-Low	Rlip-High	-logp
**All**	3951	61	34	**10.5**	1402	124	82	1.4
**Luminal androgen receptor**	203	30	12	**8.7**	83	52	16	1.9
**Her2 +**	252	32	12	**5.4**	129	72	35	1.3
**Luminal A**	1933	93	51	**4.9**	611	151	99	1.1
**Basal 1**	171	39	16	**4.0**	58	n.a.	n.a.	1.8
**Luminal B**	1149	49	35	**3.9**	433	97	70	1.1
**LN +**	1133	171	122	2.6	313	56	36	1.5
**Any chemotherapy**	798	55	36	2.6	300	n.a.	n.a.	1.5
**Grade 3**	903	37	28	2.1	503	64	45	1.8

Analysis results shown in this table were carried out on the dataset generated from the Affy_2002844_s_at microarray using KMPlotter (https://kmplot.com/analysis/index.php?p=service&cancer=breast). After removing redundant samples, 3951 cases were available for analysis. Biased arrays were excluded. A proportionality assumption was calculated in all analyses. Multivariate analysis included MKI67, ESR1, and ERRB2 for quality control. Cutoff was set at auto-select, computing all possible cutoff values between upper and lower quartile and the best performing value was used. A *p*-value as well as false discovery rate is reported. FDR was <5% for all −logp >3 (highlighted in bold). The corresponding survival curves are shown in Supplemental Data S8–S10.

## Supplementary Materials

The following are available online at https://www.mdpi.com/2072-6694/12/6/1446/s1, Figure S1: (A) Colony-forming assay for cell survival was performed with MCF7 cells per the Materials and Methods, with survival measured at 10 days after treatment. (B,C) Targeting Rlip with antibodies regressed MCF7 breast tumor growth in vivo. MCF7 cells were implanted subcutaneously on one flank of Hsd:athymic nu/nu female mice 5 days after subcutaneous implantation of estrogen pellets. Mice were treated with 200 μg of Rlip antibodies or with 200 μg/100 μL of pre-immune serum. Treatment was started when the cross-sectional area of the tumor was >42 mm2. (D) Immunostains of MCF7 tumor tissue against Ki67, CD31, and E-cadherin as well as the corresponding quantified relative staining intensity. Figure S2: (A,B) Dot plot of tumor growth and study design of MCF7 xenograft. (C,D) Dot plot of tumor growth and study design of MDA-MB-231luc xenograft. Figure S3: The effect of Rlip depletion on body weight change in mice bearing MCF7 or MDA-MB-231 tumor xenograft. Data S1: (A) Summary of all cases in METABRIC data from cBioportal. (B) Summary restricted to cases in which Rlip expression was increased in RNA-Seq analyses with z-score cutoff of 1.3 in the METABRIC data from cBioportal. (C) Summary restricted to cases in which Rlip expression was decreased in RNA-Seq analyses with z-score cutoff of -1.3. from METABRIC from cBioportal. (D) Summary restricted to cases in which Rlip expression was in the top quartile in microarray analyses in the METABRIC data from cBioportal. (E) Summary restricted to cases in which Rlip expression was in the bottom quartile in microarray analyses in the METABRIC data from cBioportal. Data S2: The Oncoprint Summary of a Query of Metabric by Alteration in Genes Differentially Expressed and with CpG Island Promoter Differentially Methylated Regions in p53-/-mice That Were Returned to Wild-type by Rlip-depletion. Data S3: Overall survival curves of Rlip-linked breast cancer genes. Data S4: Amplifications outnumber deletions in breast cancer and amplification frequency across all amplified genes varies with Rlip level. Data S5: CNA frequency across all breast cancer genes with CNAs in the Metabric breast cancer database. Data S6: CNA frequency across all breast cancer genes with CNAs in the Metabric breast cancer database. Data S7: Mutation frequency across the breast cancer genes with >5% frequency of any mutation in the Metabric breast cancer database. Data S8: Overall and relapse-free survival curves of subsets of breast cancers with respect to Rlip expression. Data S9: Overall and relapse-free survival curves of subsets of breast cancers with respect to Rlip expression. Data S10: Overall and relapse-free survival curves of subsets of breast cancers with respect to Rlip expression. Data S11: (A) Direct Interaction Network of Top Altered Genes in Breast Cancer Overlaid with Differentially Expressed Genes in p53-/-vs. wild-type C57Bl/6 mouse liver. (B) Direct Interaction Network of Top Altered Genes in Breast Cancer Overlaid with Differentially Expressed Genes in Rlip antisense-treated p53-/-vs. wild-type C57Bl/6 mouse liver. (C) Direct Interaction Network of Top Altered Genes in Breast Cancer Overlaid with Differentially Expressed Genes in Rlip+/-vs. wild-typeC57Bl/6 mouse liver. Data S12: Rlip antibody specificity was established in cultured mouse embryonic fibroblasts (MEFs) (WT and Rlip KO) by electron microscopy. Data S13: Effect of Rlip-LNA on DNA fragmentation in MDA-MB-231 and MCF7 cells 12 h after treatment with CAS or Rlip-LNA. Data S14: Representative photomicrographs of hematoxylin and eosin (H&E) staining of tumor sections. Data S15: Supplemental data for Fig. 2A. Rlip (RalBP-1) knockdown in MDA-MB-231 and MCF-7 cells after transfection with RlipLNAfor 24h as shown by western blot analysis. Data S16: supplemental data for Fig. 8. Numbers beneath each blot represent protein band intensity ratios after normalization against β-Actin. Data S17: Supplemental data for Figure 8. Effect of Rlip-LNA on signaling proteins of tumors from MCF7 and MDA-MB-231luc cell line xenograft. Representative unprocessed western blots. Data S18: Supplemental data for Figure 8. Effect of Rlip-LNA on signaling proteins of tumors from MCF7 and MDA-MB-231luc cell line xenograft. Representative unprocessed western blots. Data S19: Supplemental data for Figure 8. Effect of Rlip-LNA on signaling proteins of tumors from MCF7 and MDA-MB-231luc cell line xenograft. Representative unprocessed western blots. Data S20: Supplemental data for Figure 8. Effect of Rlip-LNA on signaling proteins of tumors from MCF7 and MDA-MB-231luc cell line xenograft. Representative unprocessed western blots.
