# Supplementary material for: Rlip Depletion Suppresses Growth of Breast Cancer
Source: Cancers (Basel). 2020 Jun 2;12(6):1446. doi: 10.3390/cancers12061446 (PMC7352702; doi:10.3390/cancers12061446)
Supplement: Supplementary file 1 [file cancers-12-01446-s001.zip › cancers-801743-suppl data.pdf]

All cases

ER+Her2+
ER- Her2-
ER+Her2low
Her2+ER-

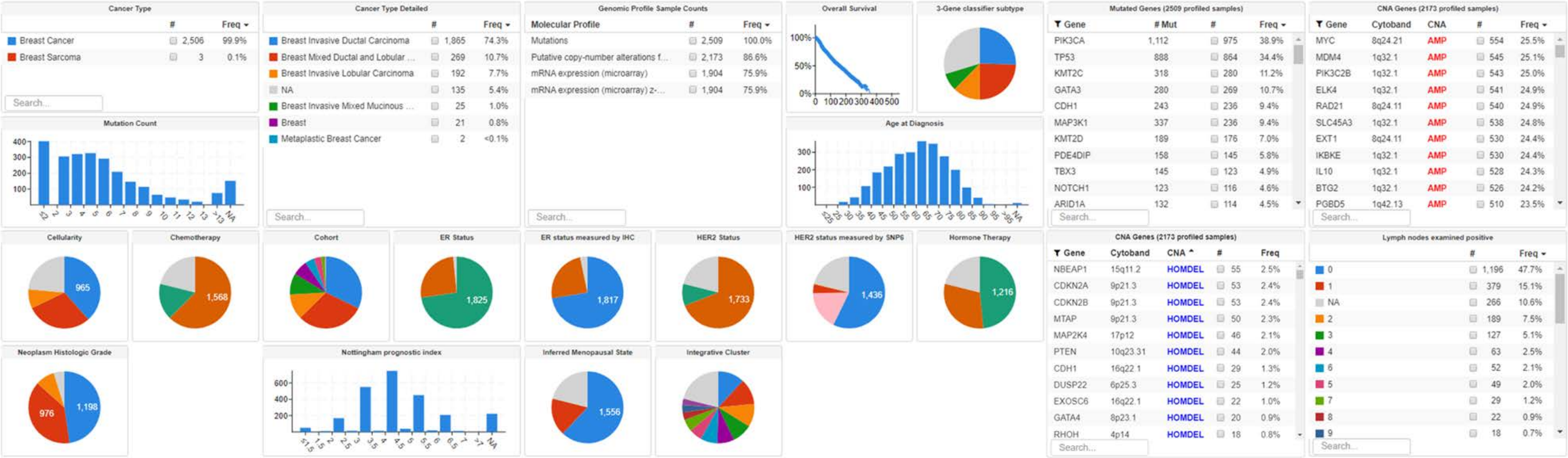

<u>Supplemental Data 1A.</u> Summary of all cases in METABRIC data from cBioportal. (https://www.cbioportal.org/study/summary?id=brca_metabric). Details of the methods and analyses have been published (Curtis C, et.al. The genomic and transcriptomic architecture of 2,000 breast tumors reveals novel subgroups. Nature. 2012 Apr 18;486(7403):346-52). The default settings of cBioportal summary was used that does not include specifics of z-score. Note the differences in gene classifier subtypes and mutation as well as CNA frequencies.

Z score >1.3 RNA-Seq

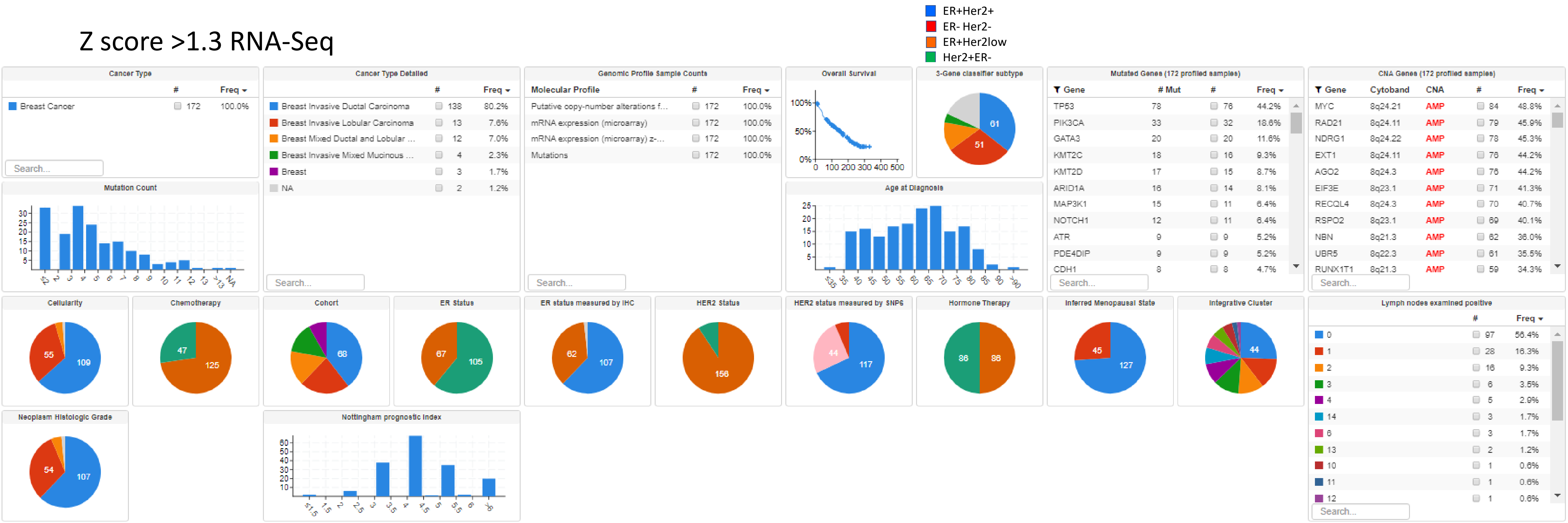

Supplemental Data. 1B. Summary restricted to cases in which Rlip expression was increased in RNA-Seq analyses with z-score cutoff of 1.3 in the METABRIC data from cBioportal. (https://www.cbioportal.org/study/summary?id=brca_metabric). Details of the methods and analyses have been published (Curtis C, et.al. The genomic and transcriptomic architecture of 2,000 breast tumours reveals novel subgroups. Nature. 2012 Apr 18;486(7403):346-52). The default settings of cBioportal summary was used and the cases selected where Rlip expression was increased as defined by z-score >1.3 (172 cases, range 1.3 – 5.97, median 1.79, average 2.42 $\pm$ 0.74).

Z score <-1.3 RNA-Seq

ER+Her2+
ER- Her2-
ER+Her2low
Her2+ER-

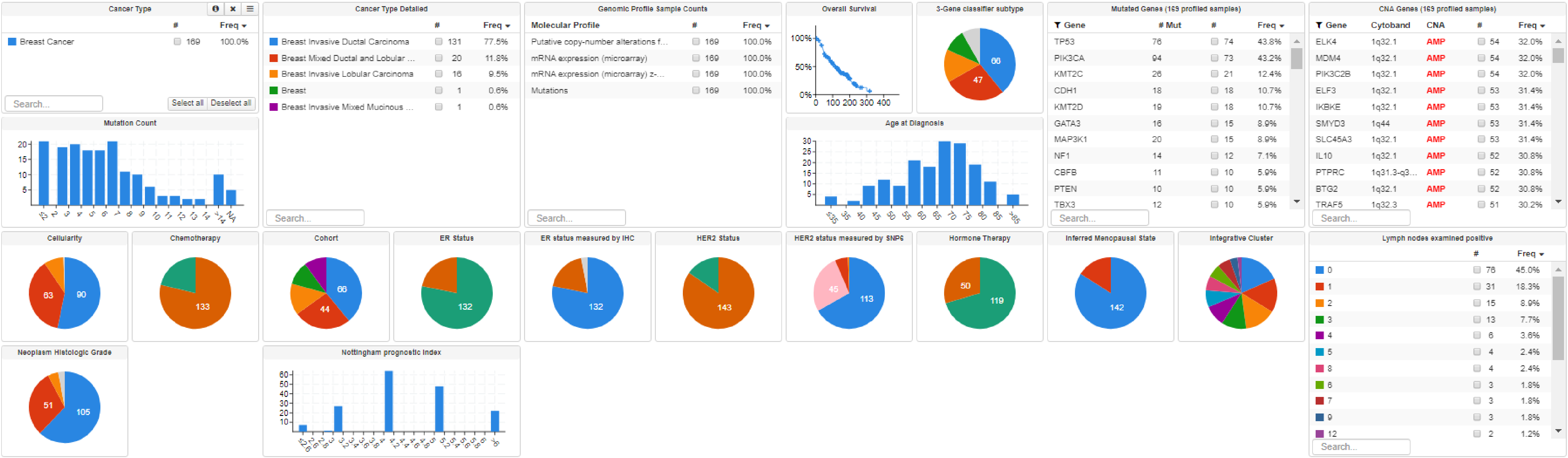

<u>Supplemental Data 1C</u>. Summary restricted to cases in which Rlip expression was decreased in RNA-Seq analyses with z-score cutoff of -1.3. from METABRIC from cBioportal. (<u>https://www.cbioportal.org/study/summary?id=brca_metabric</u>). Details of the methods and analyses have been published (Curtis C, et.al. The genomic and transcriptomic architecture of 2,000 breast tumours reveals novel subgroups. Nature. 2012 Apr 18;486(7403):346-52). The other settings of cBioportal summary were as per default was used and the cases selected where Rlip expression was decreased as defined by z-score < -1.3 (169 cases, range 1.3 – 5.97, median 1.79, average -1.71 ± 0.37).

Top Quartile (gene-expression microarray)

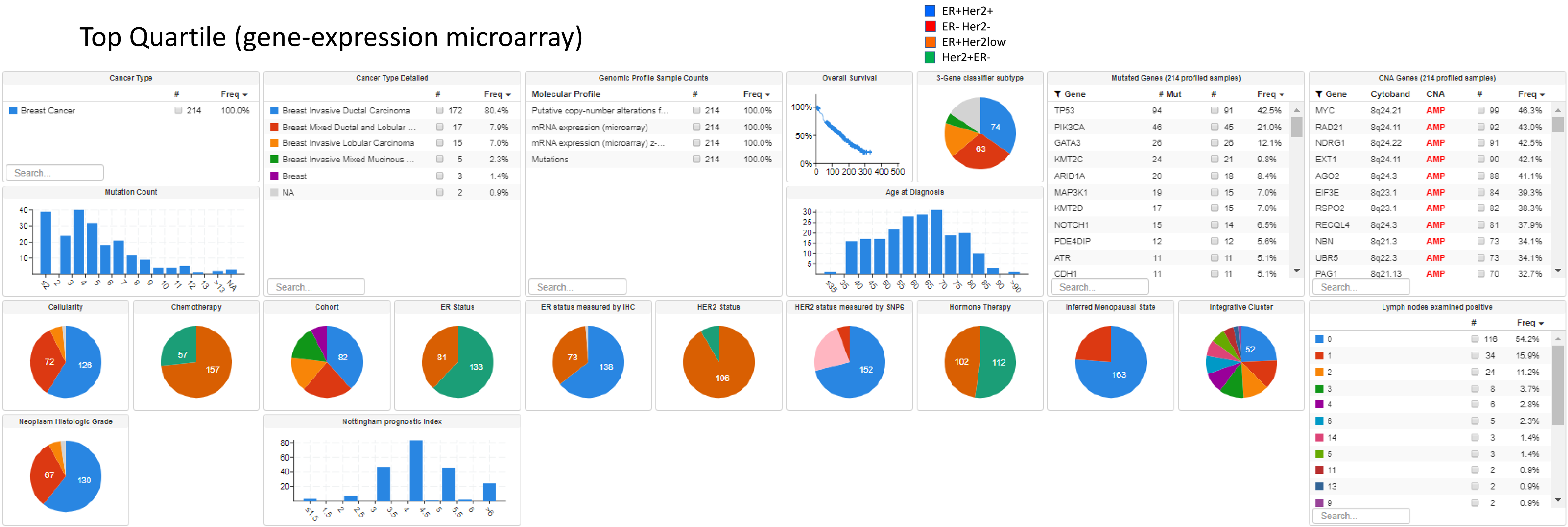

<u>Supplemental Data 1D</u>. Summary restricted to cases in which Rlip expression was in the top quartile in microarray analyses in the METABRIC data from cBioportal. (<u>https://www.cbioportal.org/study/summary?id=brca_metabric</u>). Details of the methods and analyses have been published (Curtis C, et.al. The genomic and transcriptomic architecture of 2,000 breast tumors reveals novel subgroups. Nature. 2012 Apr 18;486(7403):346-52). The default settings of cBioportal summary was used and the cases selected where Rlip expression was in the top quartile (214 cases, range 7.80-9.64, median 7.98, average 8.04 ± 0.23).

Bottom Quartile (gene-expression microarray)

ER+Her2+
ER- Her2-
ER+Her2low
Her2+ER-

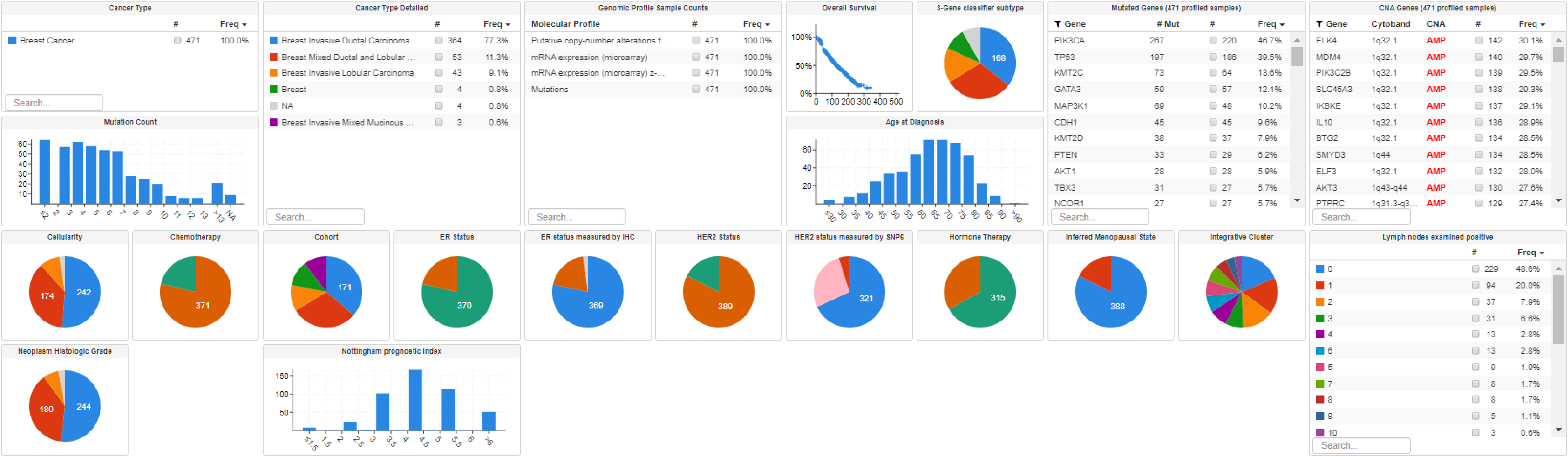

<u>Supplemental Data 1E</u>. Summary restricted to cases in which Rlip expression was in the bottom quartile in microarray analyses in the METABRIC data from cBioportal. (<u>https://www.cbioportal.org/study/summary?id=brca_metabric</u>). Details of the methods and analyses have been published (Curtis C, et.al. The genomic and transcriptomic architecture of 2,000 breast tumours reveals novel subgroups. Nature. 2012 Apr 18;486(7403):346-52). The default settings of cBioportal summary was used and the cases (74) selected where Rlip expression was in the bottom quartile (471 cases, range 6.35-7.23, median 7.38, average 7.18 $\pm$ 0.16).

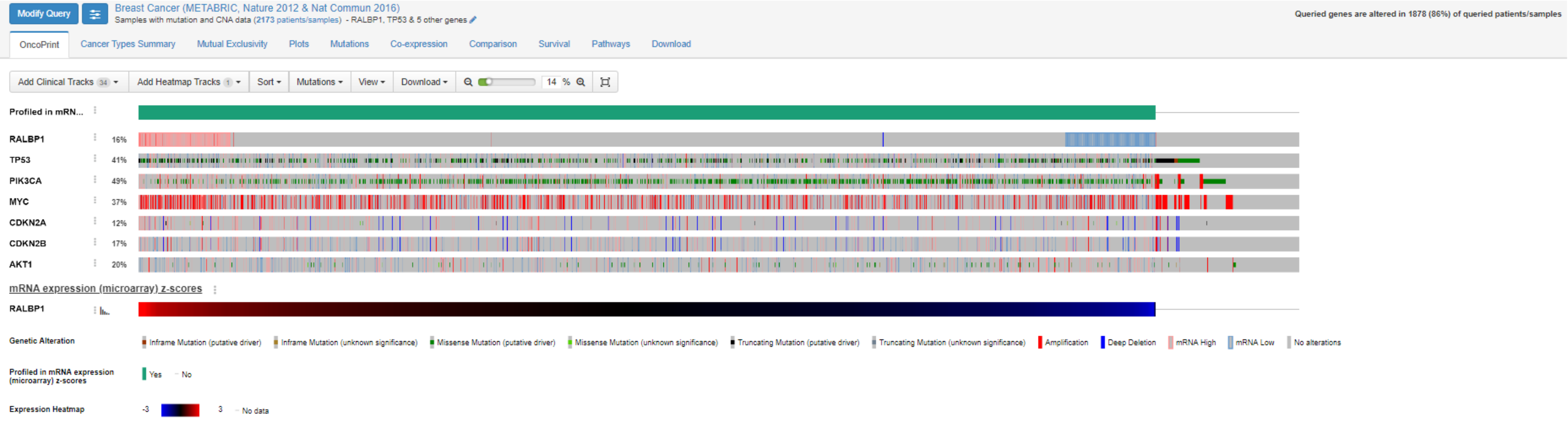

<u>Supplemental Data 2</u>. The Oncoprint Summary of a Query of Metabric by Alteration in Genes Differentially Expressed and with CpG Island Promoter Differentially Methylated Regions in p53-/- mice That Were Returned to Wild-type by Rlip-depletion. The query design was as follows:
(https://www.cbioportal.org/results/oncoprint?Action=Submit&<u>RPPA_SCORE_THRESHOLD=2.0&Z_SCORE_THRESHOLD=1.3</u>&cancer_study_list=brca_metabric&case_set_id=brca_metabric_cnaseq&data_priority=0&gene_list=RALBP1%250ATP53%250APIK3CA%250AMYC%250ACDKN2A%250ACDKN2B%250AAKT1&geneset_list=%20&genetic_profile_ids_PROFILE_COPY_NUMBER_ALTERATION=brca_metabric_cna&genetic_profile_ids_PROFILE_MRNA_EXPRESSION=brca_metabric_mrna_median_Zscores&genetic_profile_ids_PROFILE_MUTATION_EXTENDED=brca_metabric_mutations&heatmap_track_groups=brca_metabric_mrna_median_Zscores%2CRALBP1&profileFilter=0&tab_index=tab_visualize).

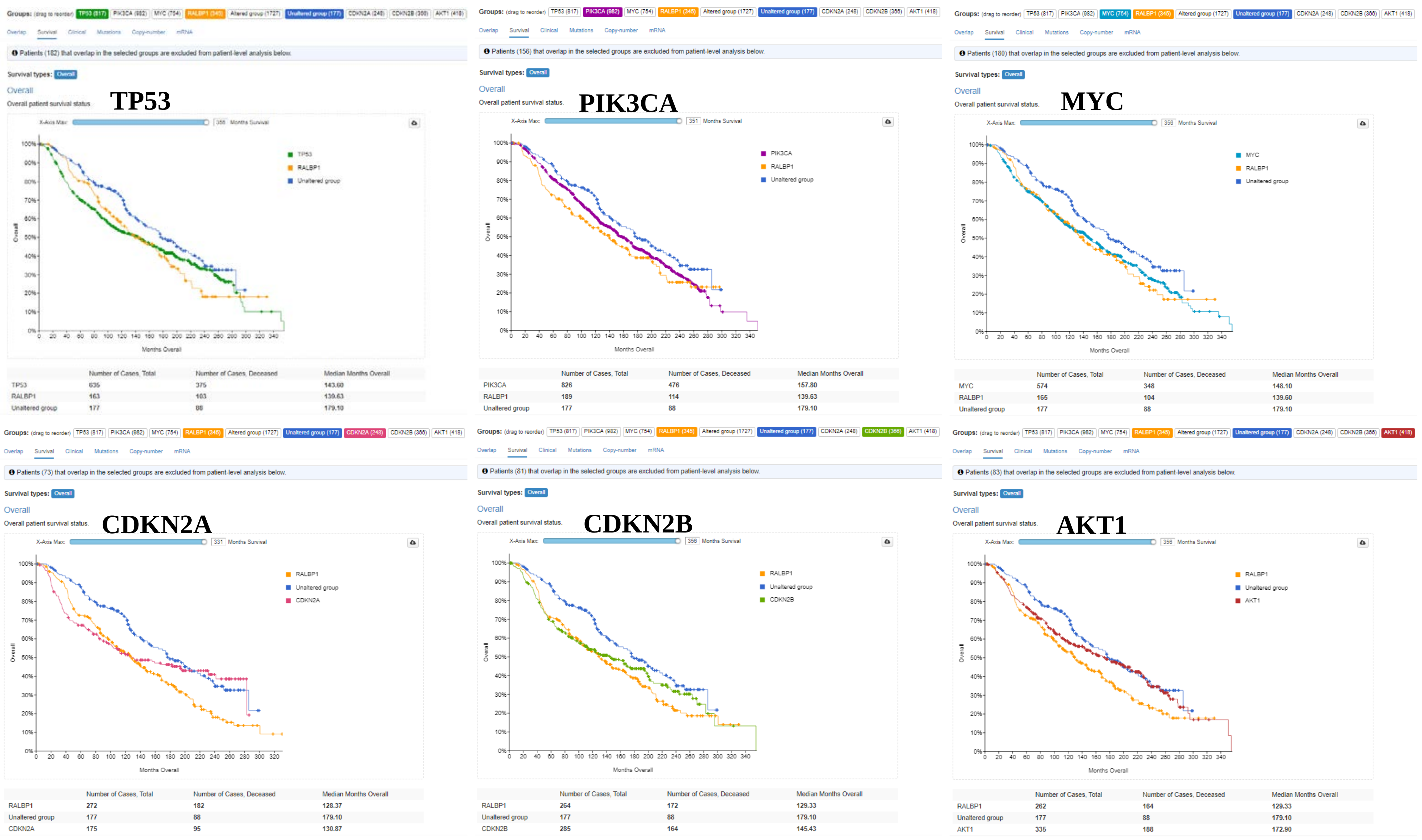

	log rank p-value
**RALBP1**	4.30E-04
**TP53**	2.01E-03
**PIK3CA**	1.31E-02
**MYC**	1.66E-03
**CDKN2A**	5.87E-02
**CDKN2B**	4.52E-03
**AKT1**	6.85E-02

<u>Supplemental Data 3</u>. Overall survival curves of Rlip-linked breast cancer genes. The survival curves presented are from the query in Fig. 3. All six genes were differentially expressd and had CPG island promoter differentially methylated regions in p53-/- mice that were returned to wild-type by Rlip-depletion. The log rank test p-values are presented in the accompanying table.

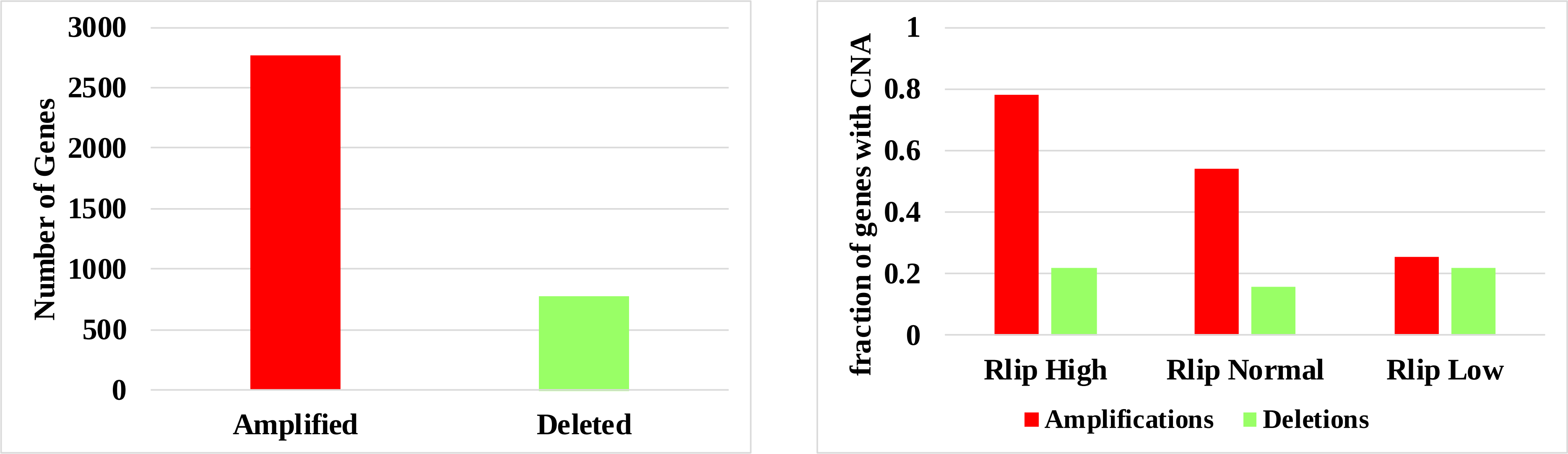

Supplemental Data 4. **Amplifications outnumber deletions in breast cancer and amplification frequency across all amplified genes varies with Rlip level.** The cBioportal website was used to download the CNA and mutation data from all breast cancer cases in Metabric. cBioportal was also used to query the Metabric database for alterations in Rlip with z-score cutoff of 1.645. The "Rlip High" subset consisted of cases in which chromosomal bands encompassing the Rlip gene were amplified or cases having increased Rlip mRNA expression as defined by the Metabric study. Similarly, the "Rlip Low" subset consisted of cases in which chromosomal bands encompassing the Rlip gene were deleted or cases having decreased Rlip mRNA expression as defined by the Metabric study.

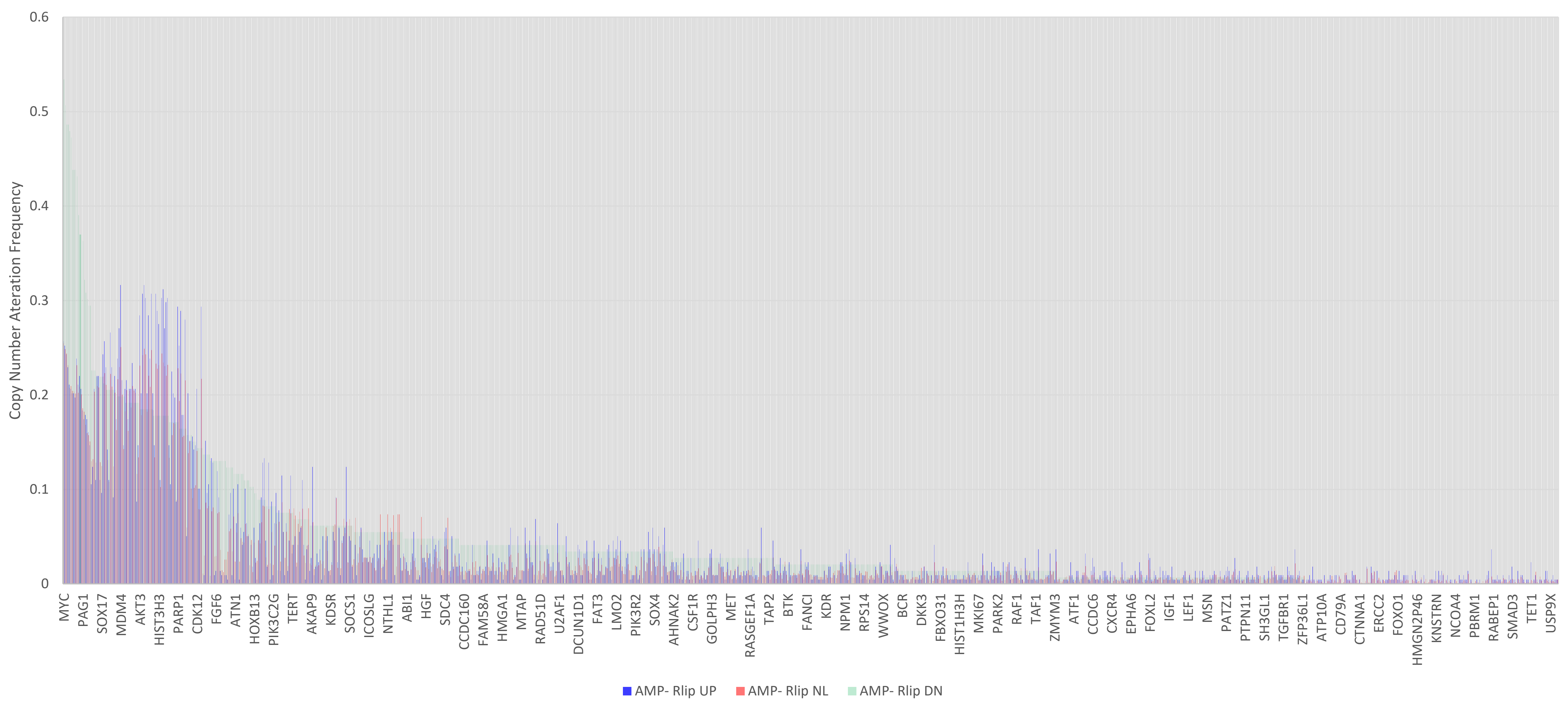

<u>Supplemental Data 5</u>. CNA frequency across all breast cancer genes with CNAs in the Metabric breast cancer database. The cases were divided into those with increased (UP, upper quartile), decreased (DN, lower quartile) and unchanged (NL, middle quartiles). Statistical analysis was performed using Prism 6.0 for Windows (GraphPad, SanDiego, CA). Data for all outcomes were summarized by group as the mean ±SD (standard deviation). The differences between Rlip-up, Rlip-low and Rlip-normal were assessed for statistical significance at alpha = 0.05 via unpaired test with Welch correction and . For the multiple comparisons between the groups One-way analysis of variance (ANOVA) with the Tukey-Kramer multiple comparison test and Kruskal-Wallis post-hoc test with DUNN's correction were performed. A P value of < 0.05 was considered statistically significant.

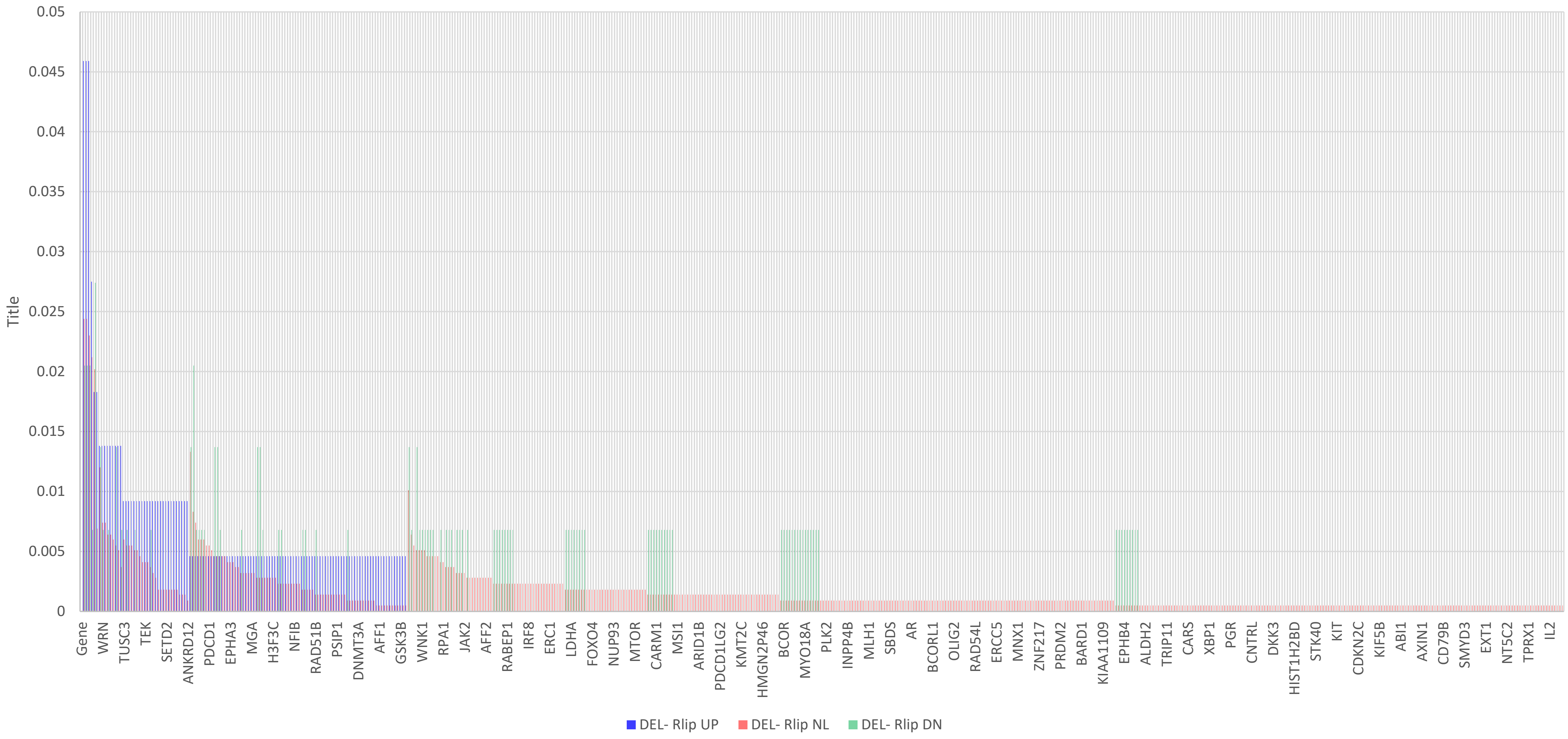

<u>Supplemental Data 6</u>. CNA frequency across all breast cancer genes with CNAs in the Metabric breast cancer database. The cases were divided into those with increased (UP, upper quartile), decreased (DN, lower quartile) and unchanged (NL, middle quartiles). (expandable by stretching the graph to see gene names)

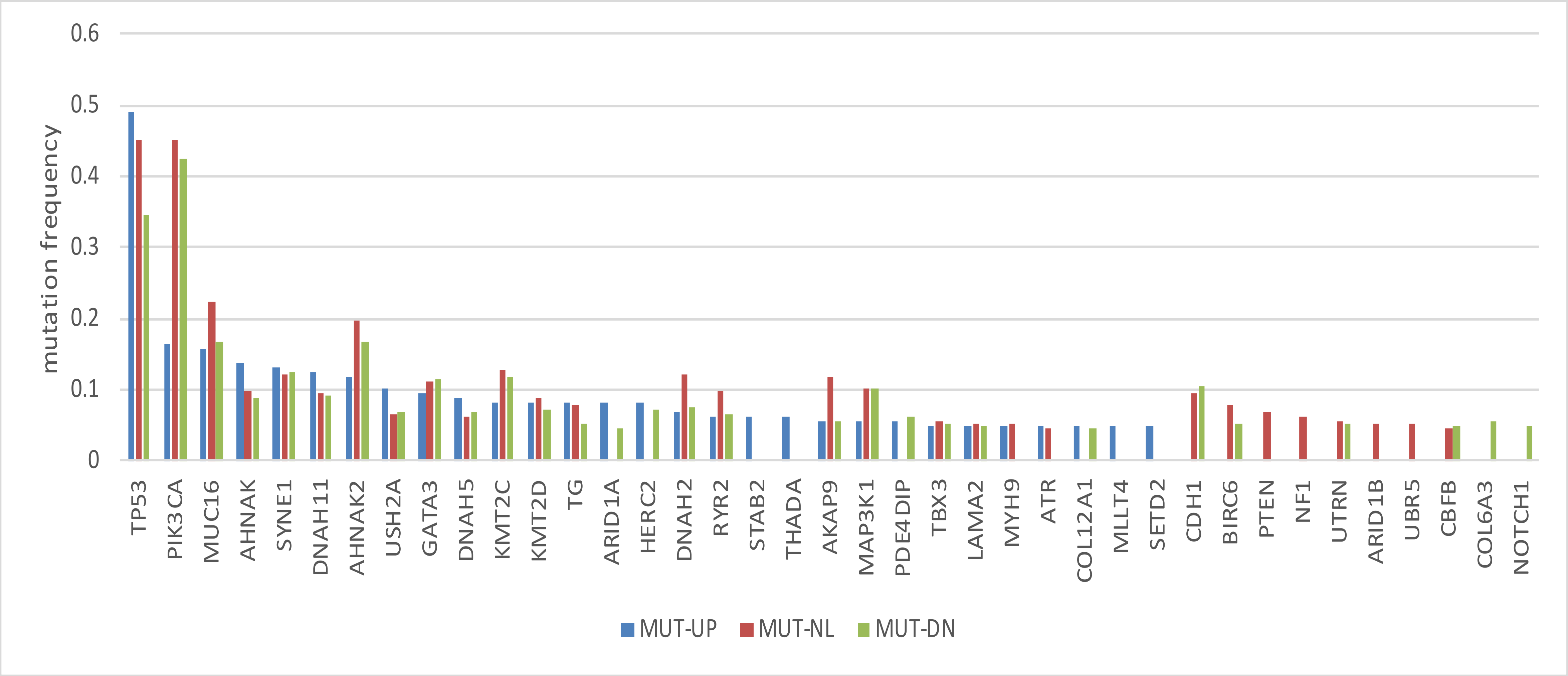

Supplemental Data 7. Mutation frequency across the breast cancer genes with >5% frequency of any mutation in the Metabric breast cancer database. The cases were divided by Rlip levels into those with increased, decreased (DN, lower quartile) and unchanged (NL, middle quartiles) levels (**Curtis C, et.al. Nature. 2012; 18;486(7403):346-52**).

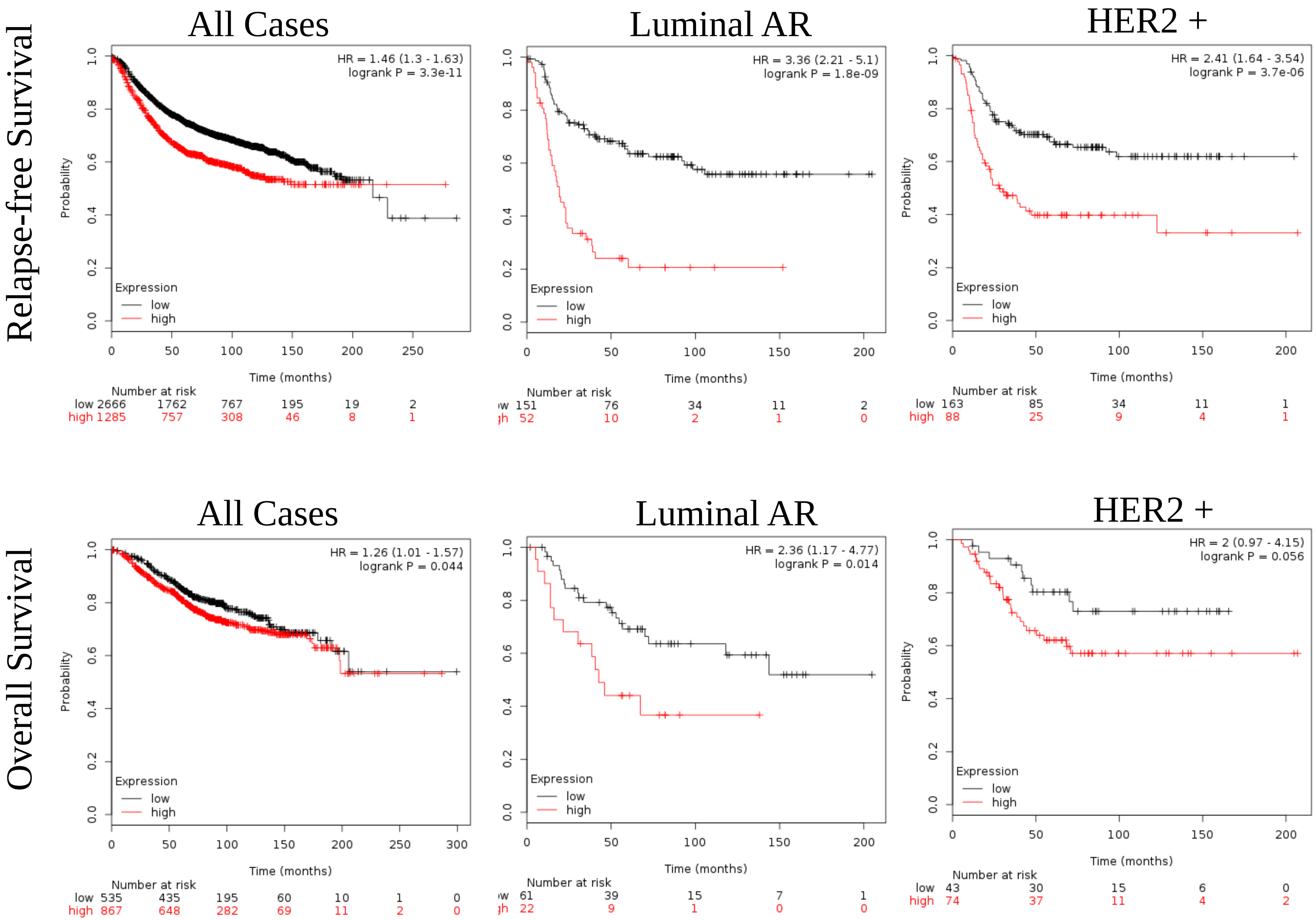

<u>Supplemental Data 8</u>. Overall and relapse-free survival curves of subsets of breast cancers with respect to Rlip expression. Details of derivation of curves are given in the legend of manuscript *Table 2.*

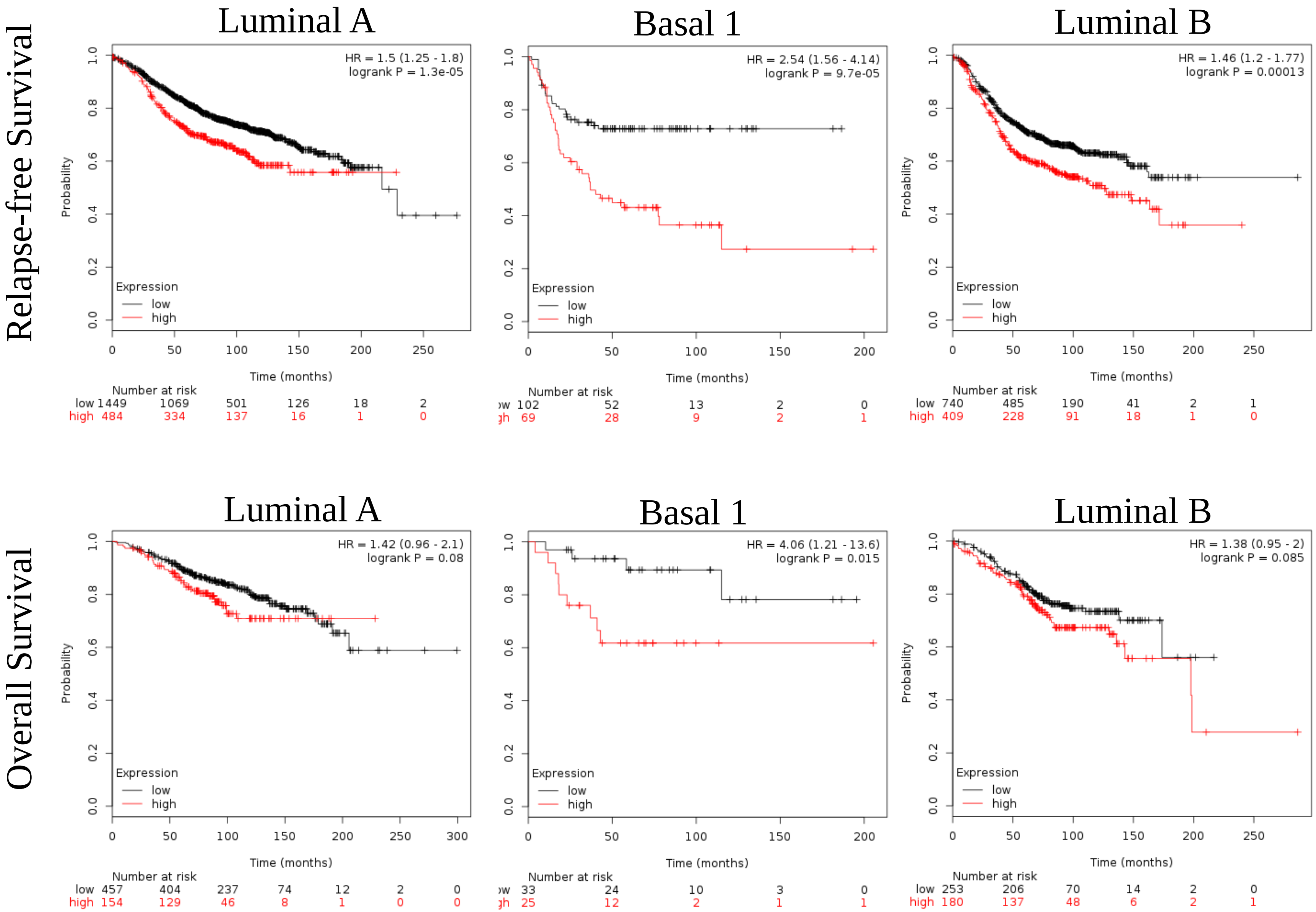

Supplemental Data 9. Overall and relapse-free survival curves of subsets of breast cancers with respect to Rlip expression. Details of derivation of curves are given in the legend of manuscript *Table 2*.

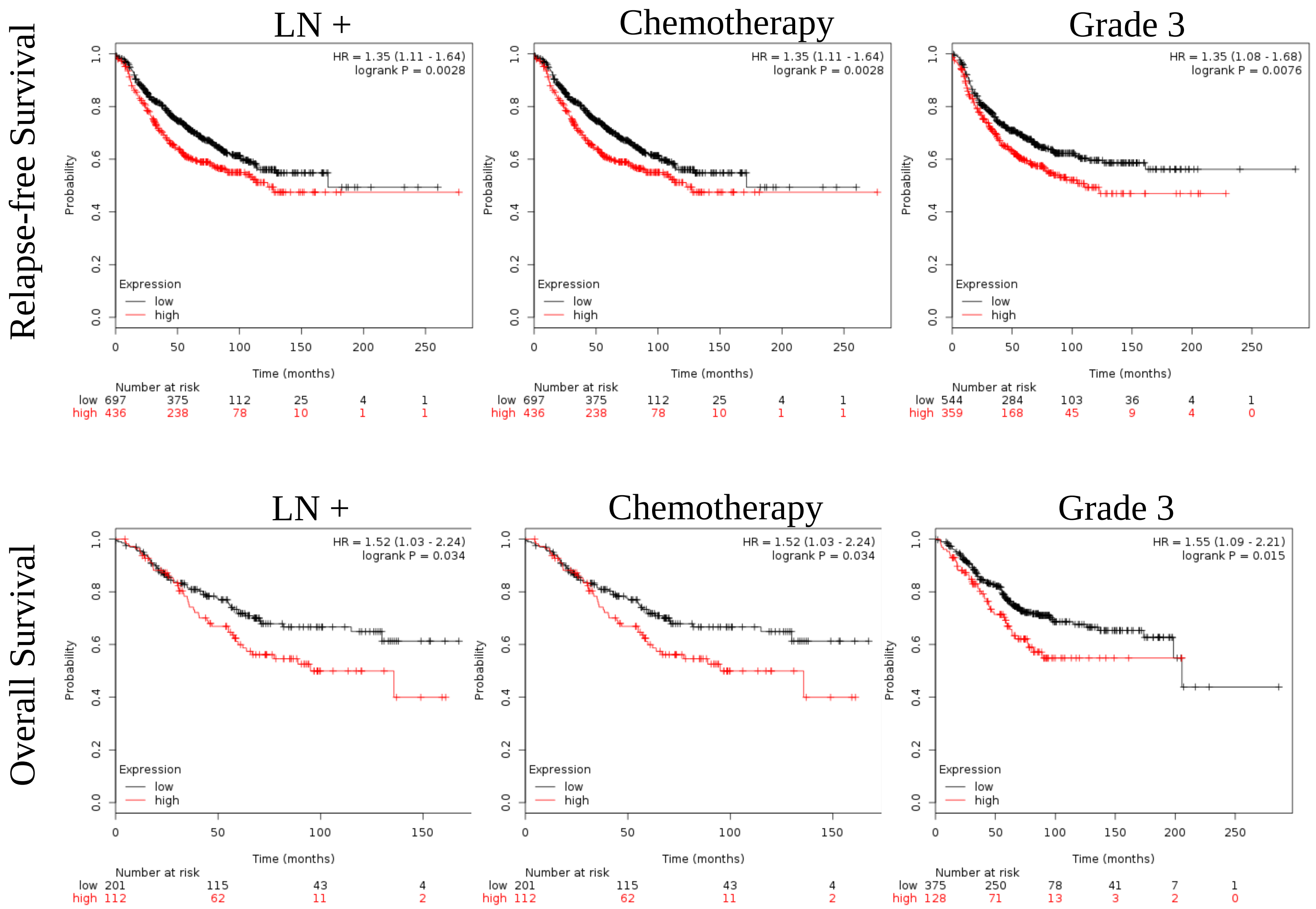

<u>Supplemental Data 10</u>. Overall and relapse-free survival curves of subsets of breast cancers with respect to Rlip expression. Details of derivation of curves are given in the legend of manuscript *Table 2*.

Supplemental Data 11 A. **Direct Interaction Network of Top Altered Genes in Breast Cancer Overlaid with Differentially Expressed Genes in $p53^{-/-}$ vs. wild-type C57Bl/6 mouse liver.**

Rationale

The cancer susceptible p53 null mice develop age-dependent epigenomic and transcriptomic abnormalities that result in increased levels of oncogenes and reduced levels of tumor suppressors (Awasthi et al., Proc. Natl. Acad. Sci 2018; 115(15):3918-3923; PMID 29572430). The abnormal expression (as well as promoter CpG island methylation) of many oncogenes and tumor suppressors are reversed when Rlip is reduced through antisense administration or crossbreeding with Rlip null mice. Because human breast cancers frequently carry somatic p53 mutations or deletions (**Metabric reference**), we conjectured that breast cancer regression upon Rlip depletion could be due to broad spectrum effects on oncogenes and tumor suppressors resembling those found either in Rlip null mice or in p53 null mice in which Rlip deficiency was induced. Thus we queried Metabric data using cBioportal to obtain the top altered genes in breast cancer and performed network analyses using IPA (Ingenuity Pathway Analysis, Qiagen) to determine whether the expression of these breast cancer genes was affected by p53 loss or Rlip deficiency in our previously published results.

Analysis Method

The list of top 176 altered genes (mutated, amplified and deleted) in the Metabric study was down-loaded from (https://www.cbioportal.org/study/summary?id=brca_metabric) and uploaded into Ingenuity Pathway Analysis (IPA) to generate the (direct) interaction network shown here. The z-score values of differentially expressed mRNA from IPA core analysis (cutoffs log-2 fold change ± 3, -logp >5, FDR >5) of p53-null (p53-/-) mice treated with control scrambled phosphorothioate antisense was overlaid on to this network. Red is increased and green is decreased control antisense treated-$p53^{-/-}$ vs. wild-type mouse (liver tissue, n=2 mice, replicate determinations).

Conclusion: The majority of genes most frequently altered in breast cancer overlapped with the set of transcriptionally altered genes in $p53^{-/-}$ mice.

Path Designer Breast Network with Rlip and TP53

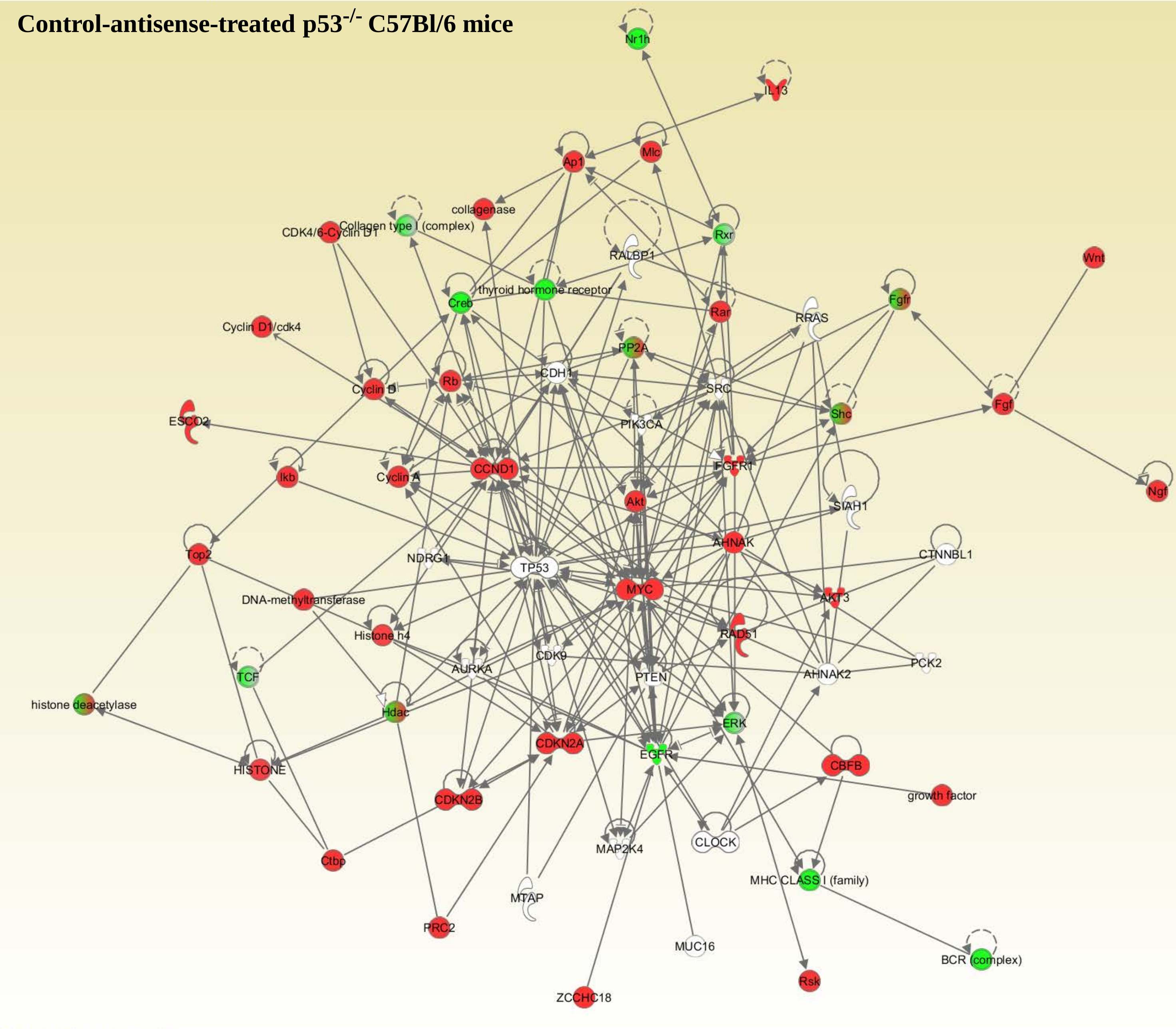

© 2000-2020 QIAGEN. All rights reserved.

Supplemental Data 11B. Direct Interaction Network of Top Altered Genes in Breast Cancer Overlaid with Differentially Expressed Genes in Rlip antisense-treated p53$^{-/-}$ vs. wild-type C57Bl/6 mouse liver. The analyses were conducted as described in Fig. 1A. The mice were injected intraperitoneally weekly with 200 µg R508 (Rlip-directed 22 nucleotide fully phosphorothioated antisense. R508 and scrambled antisense were prepared under GLP conditions in the City of Hope Nucleotide Synthesis Core Facility. Full experimental details have been published previously (PMID 29572430). Rlip protein level was ~50% of normal at the time of necropsy.

Conclusion: The direction of change in majority of breast cancer genes in the direct interaction network was opposite that observed in control p53$^{-/-}$ mice that were treated with an equal amount of control scrambled antisense.

Path Designer Breast Network with Rlip and TP53

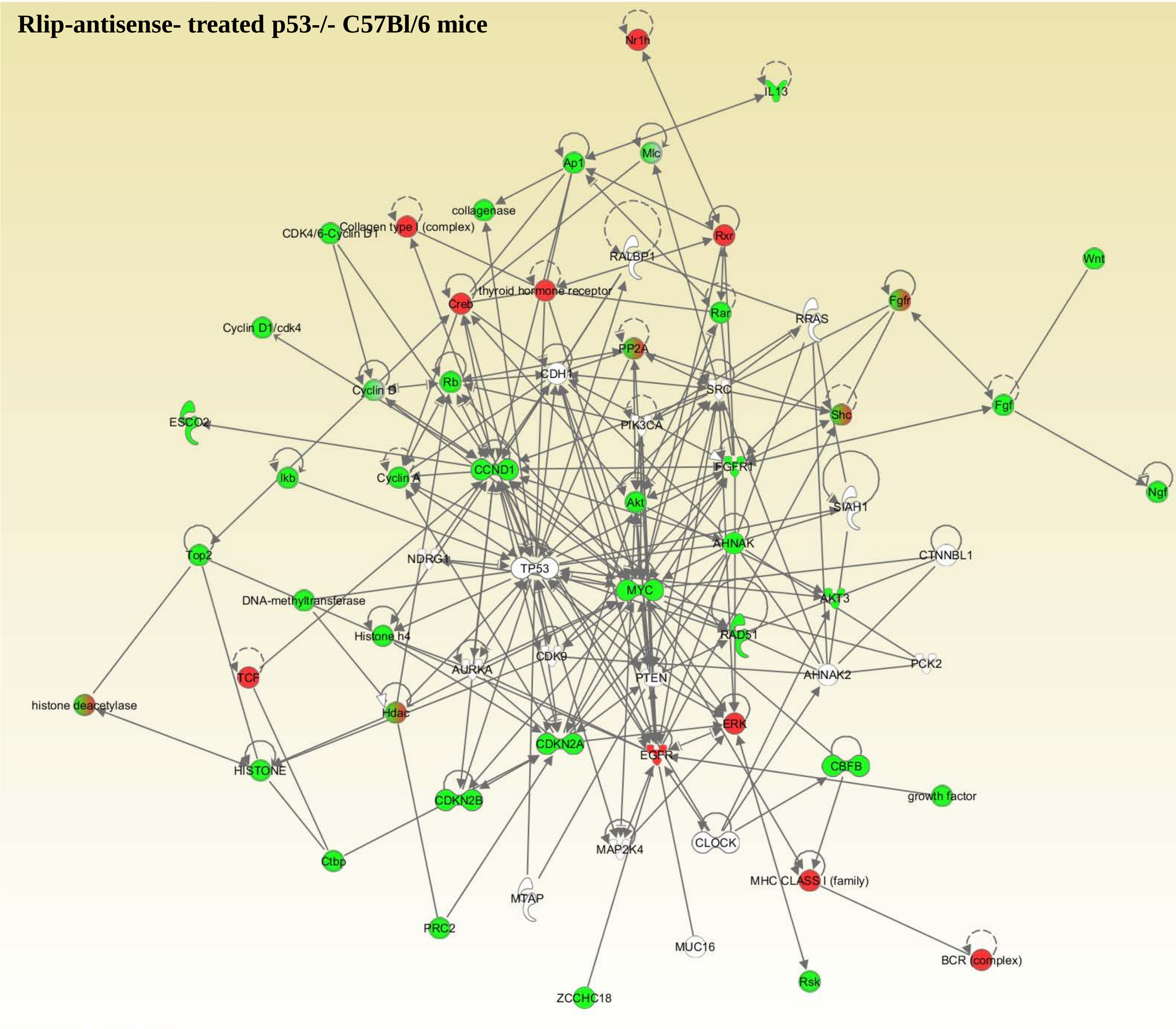

© 2000-2020 QIAGEN. All rights reserved.

Supplemental Data 11C. Direct Interaction Network of Top Altered Genes in Breast Cancer Overlaid with Differentially Expressed Genes in Rlip$^{+/-}$ vs. wild-type C57Bl/6 mouse liver. RNA expression data for Rlip knockout mice was from Affymetrix HG-U133 Plus 2.0 microarray. For Affymetrix array analyses, total RNA was isolated from fresh mouse liver tissues collected from wt, Rlip+/- or Rlip-/- mice at the time of euthanasia using Trizol (Invitrogen Life Technologies, Carlsbad, CA), purified using RNeasy columns (Qiagen, Valencia, CA) from, and stored at −80 °C until use. Biotinylated cRNA was synthesized from total RNA (Enzo, Farmingdale, NY). Following processing according to the Affymetrix GeneChip Expression Analysis Technical Manual (Affymetrix, Santa Clara, CA), labeled cRNA was quantified by hybridization to Affymetrix U133plus2.0 GeneChip analysis was performed in the Affymetrix core facility, U.T. Southwestern Medical Center, Dallas, TX. Affymetrix data was normalized and analyzed by the RMA method in the Bioconductor's 'affy' package. The manufacturer's annotations were obtained from the NetAffx web site. Two biological replicates with two technical replicates were analyzed at three dilutions.

Conclusion

The direction of change in majority of breast cancer genes in the direct interaction network was opposite that observed in control p53$^{-/-}$ mice that were treated with an equal amount of control scrambled antisense.

Path Designer Breast Network with Rlip and TP53

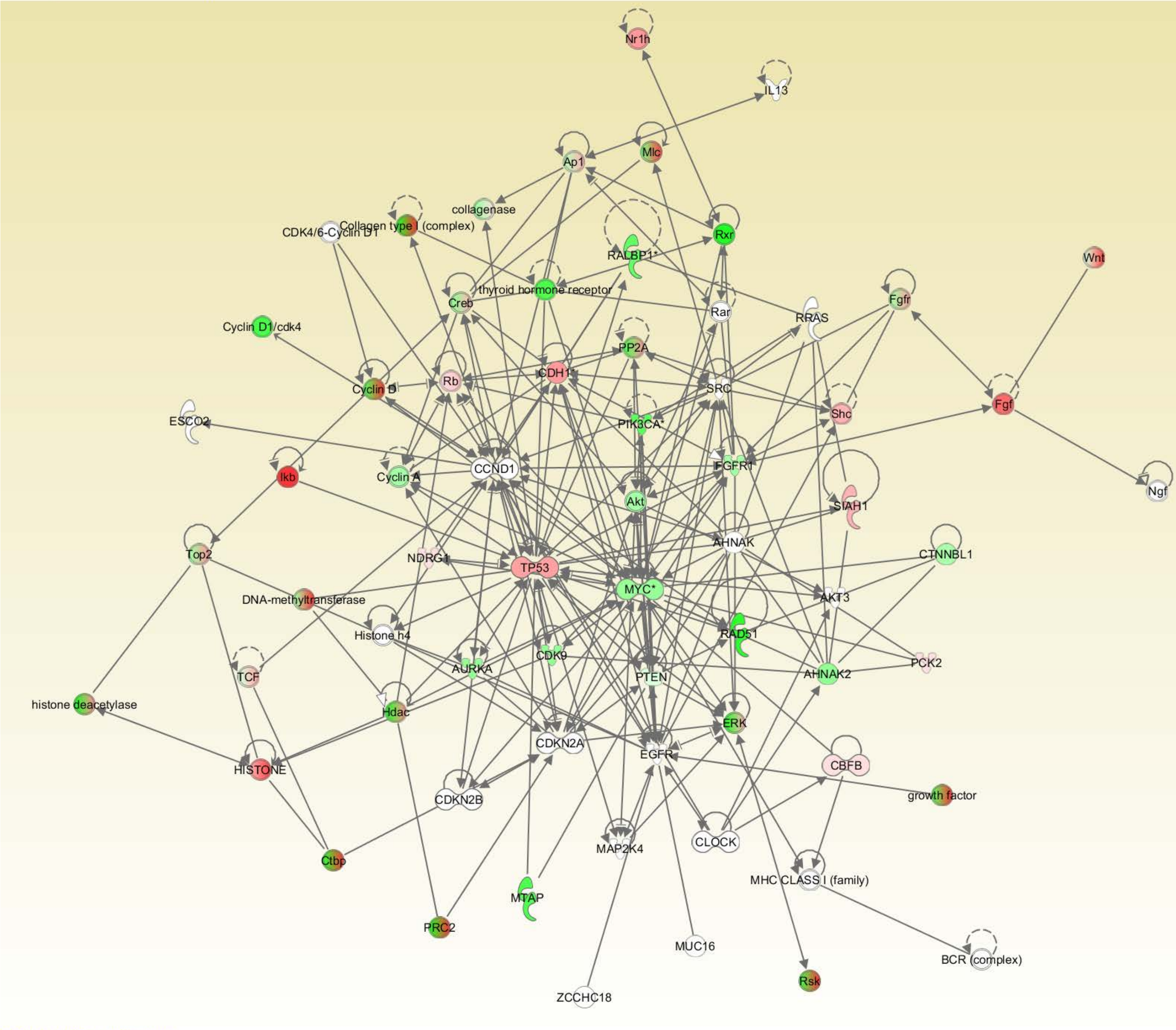

© 2000-2020 QIAGEN. All rights reserved.

Supplemental Data 12.

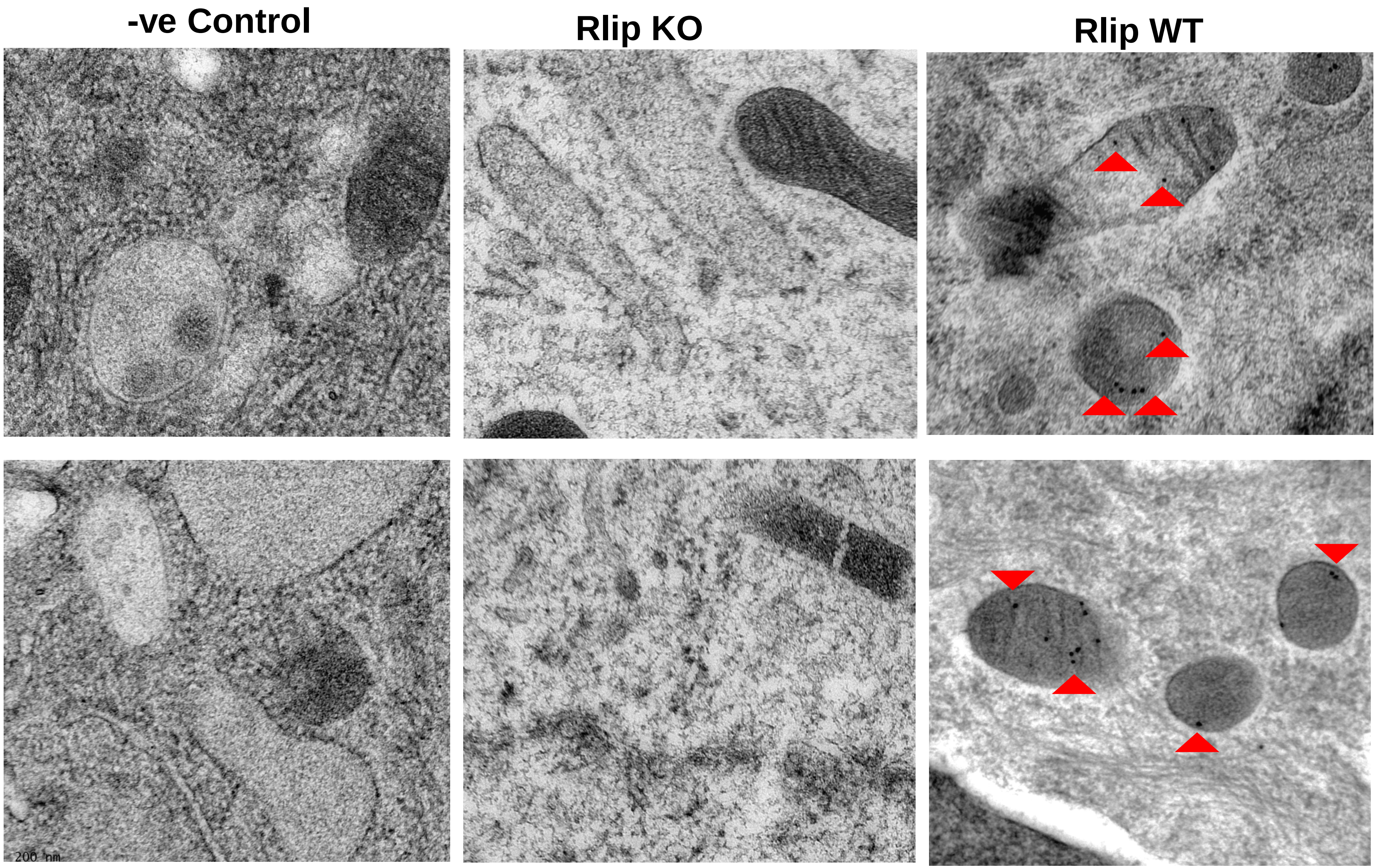

Rlip antibody specificity was established in cultured mouse embryonic fibroblasts (MEFs) (WT and Rlip KO) by electron microscopy using the method described in Materials and Methods section 4.11. Red arrow heads point to staining of Rlip.

Supplemental Data 13.

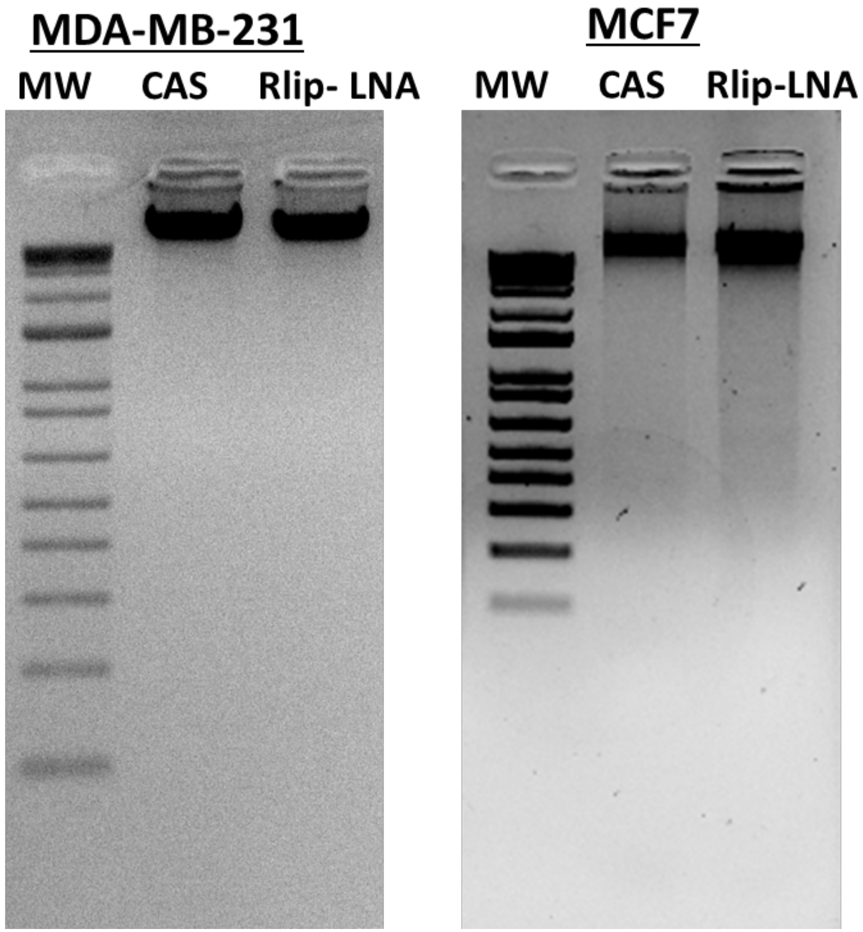

Effect of Rlip-LNA on DNA fragmentation in MDA-MB-231 and MCF7 cells 12 h after treatment with CAS or Rlip-LNA. Only MCF7 cells show signs of initiation of DNA fragmentation after 12h of the treatment.

Supplemental Data 14.

Control Tumor section

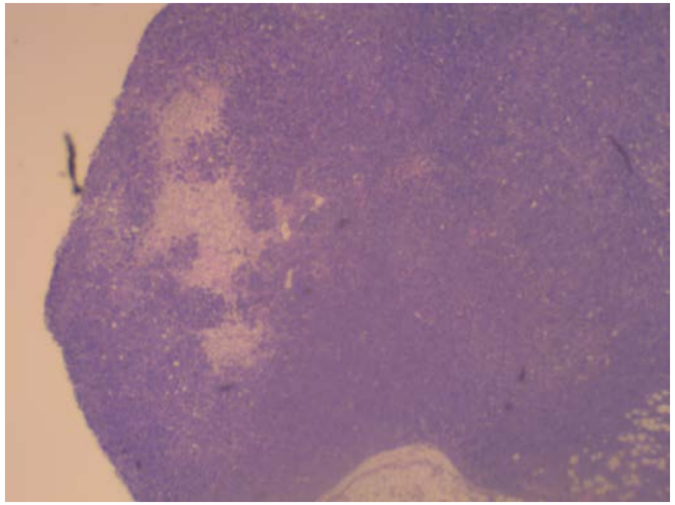

Rlip-LNA treated Tumor section

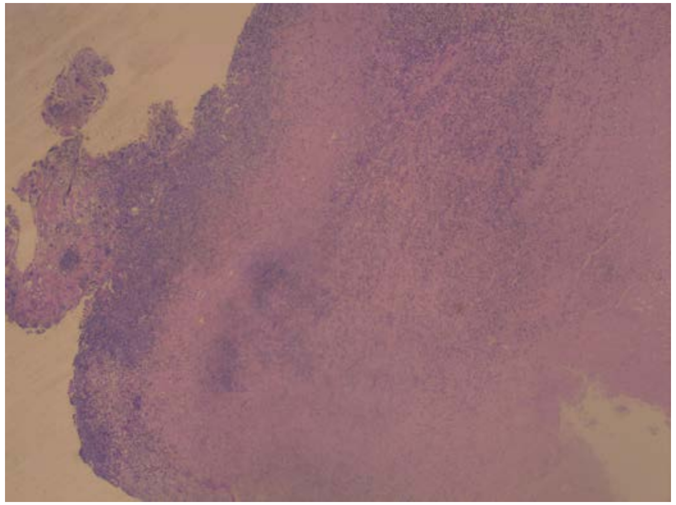

Representative photomicrographs of hematoxylin and eosin (H&E) staining of tumor sections. Control H&E-stained sections of dissected tumors show the intact tumor cell structure. Tumors treated with Rlip-LNA show small residual treated nodule is largely necrotic, but does have a rim of clearly identifiable tumor cells.

Supplemental Data 15.

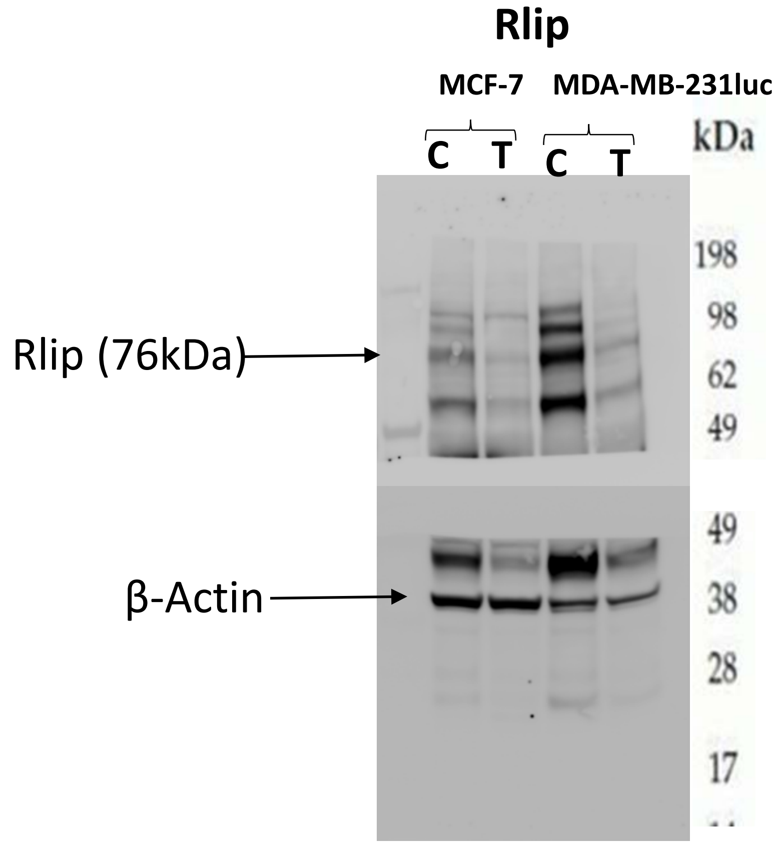

Supplemental data for Fig. 2A. Rlip (RalBP-1) knockdown in MDA-MB-231 and MCF-7 cells after transfection with RlipLNA for 24h as shown by western blot analysis. 30ug total protein was loaded on 4-12% bis-tris gel (Invitrogen) and run usingMES gel running buffer. RalBP-1 (Santacruz Biotechnology) 1:1000 O/N in 4^{0}C, secondary 1:2000, 1h at room temperature. Blocking and antibody dilution buffer used 1Xclear milk with 0.05% tween -20.

Numbers below show quantified Rlip expression after normalization against β-Actin.

C=CAS

T=Rlip-LNA

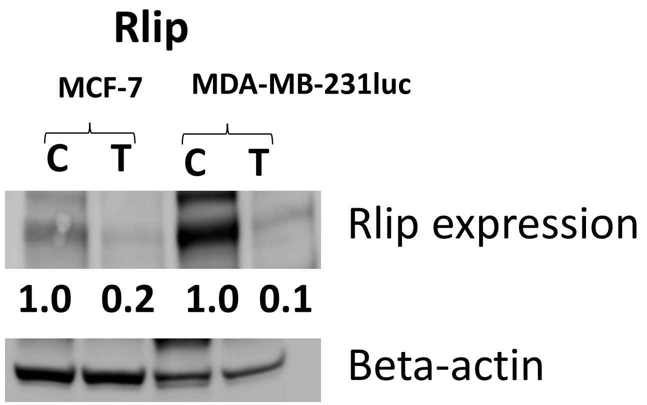

Supplemental Data 16.

Supplemental data for Fig. 8.

Numbers beneath each blot represent protein band intensity ratios after normalization against β-Actin. Full blots are presented in the next 4 figures. Intracellular signaling markers in MCF7 and MDA-MB-231luc cell induced tumors after treatment with Rlip-LNA as described in Methods section of the manuscript. Total tissue lysates were loaded on 4-12% bis-tris or tris acetate gel and resolved using 1X MES gel running buffer. Proteins were transferred to nitrocellulose membrane, nonspecific binding was done in 1X clear milk, +0.1%T20 (Invitrogen) for 1h at room temperature. All primary antibodies were from Santa Cruze Biotechnology (1:1000), in 1X clear milk +0.1%T20 overnight in 4^0C. Secondary antibody from Santacruze Biotech (1:2000) for 1h at room temperature in 1X clear milk, 0.1% T20.

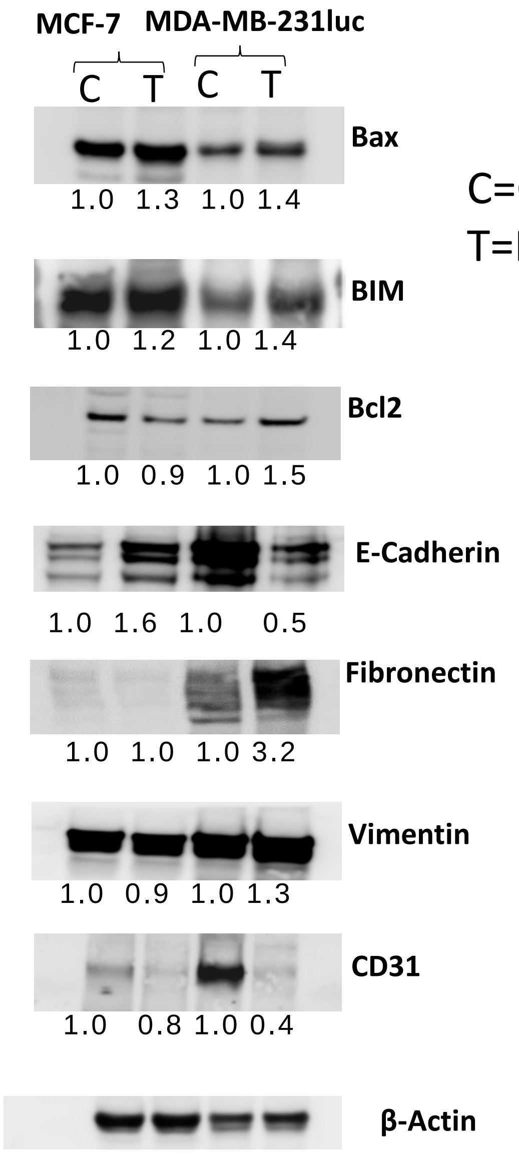

C=CAS
T=Rlip-LNA

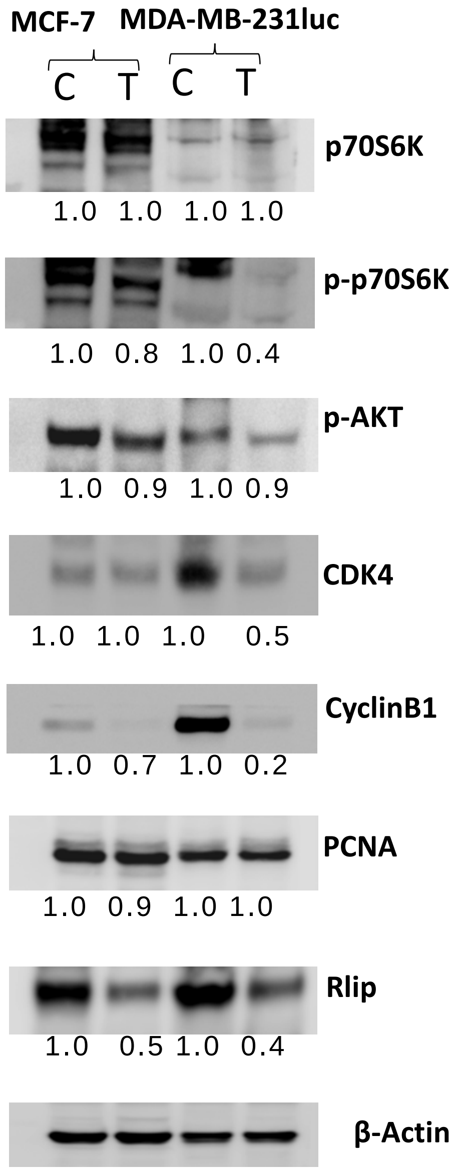

Supplemental Data 17. Full gel images

Supplemental data for Figure 8. Effect of Rlip-LNA on signaling proteins of tumors from MCF7 and MDA-MB-231luc cell line xenograft. Representative unprocessed western blots of control (C) and Rlip-LNA (T) treated animal tumors as shown in figure 8. Red Arrow indicate cropped area included in figure 8.

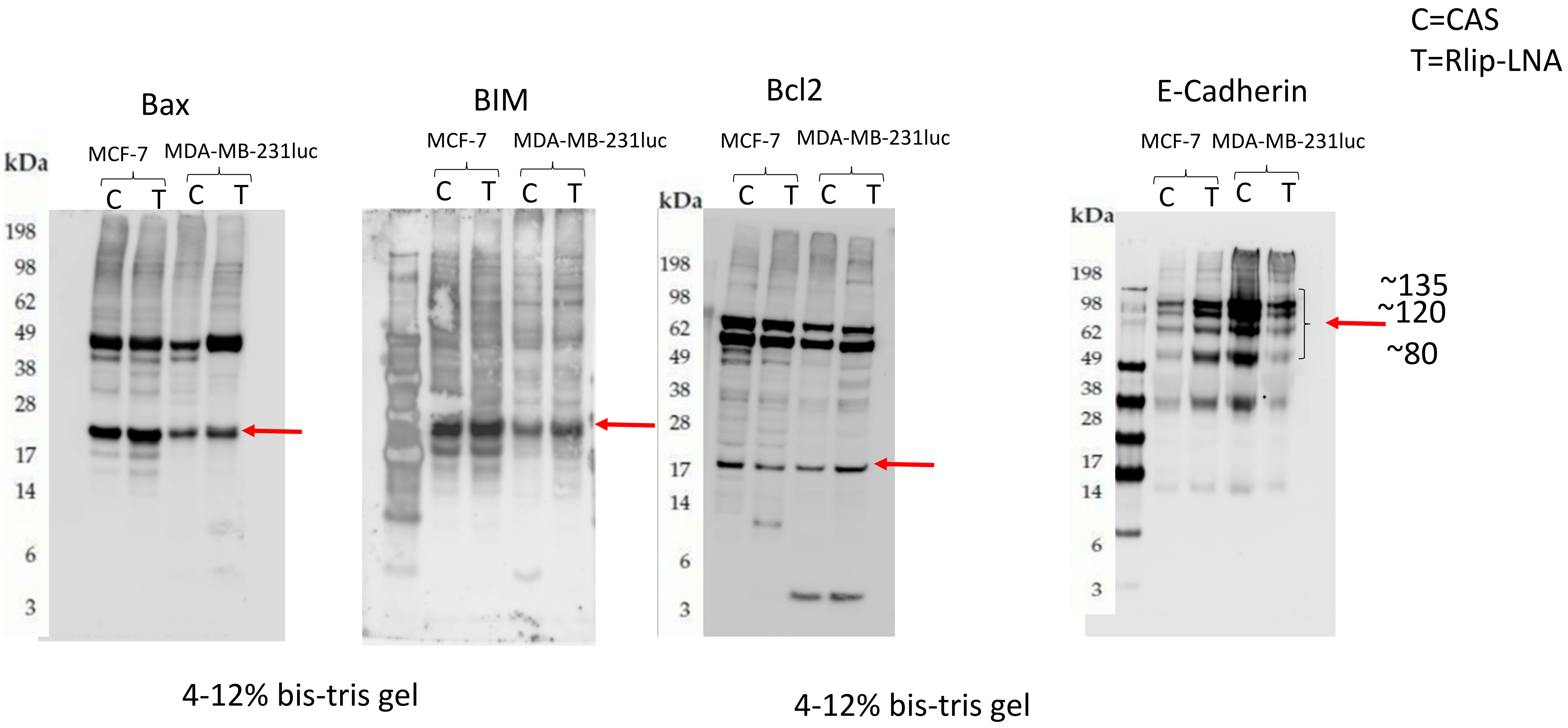

Supplemental Data 18.

Supplemental data for Figure 8. Effect of Rlip-LNA on signaling proteins of tumors from MCF7 and MDA-MB-231luc cell line xenograft. Representative unprocessed western blots of control (C) and Rlip-LNA (T) treated animal tumors as shown in figure 8. Red Arrow indicate cropped area included in figure 8.

C=CAS

T=Rlip-LNA

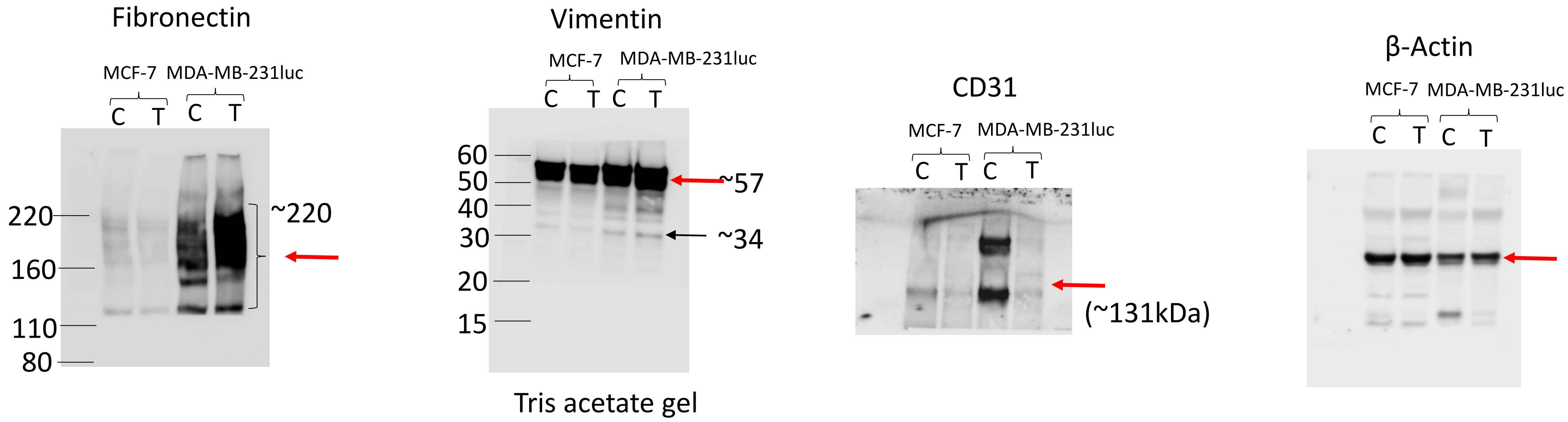

Supplemental Data 19.

Supplemental data for Figure 8. Effect of Rlip-LNA on signaling proteins of tumors from MCF7 and MDA-MB-231luc cell line xenograft. Representative unprocessed western blots of control (C) and Rlip-LNA (T) treated animal tumors as shown in figure 8. Red Arrow indicate cropped area included in figure 8.

C=CAS

T=Rlip-LNA

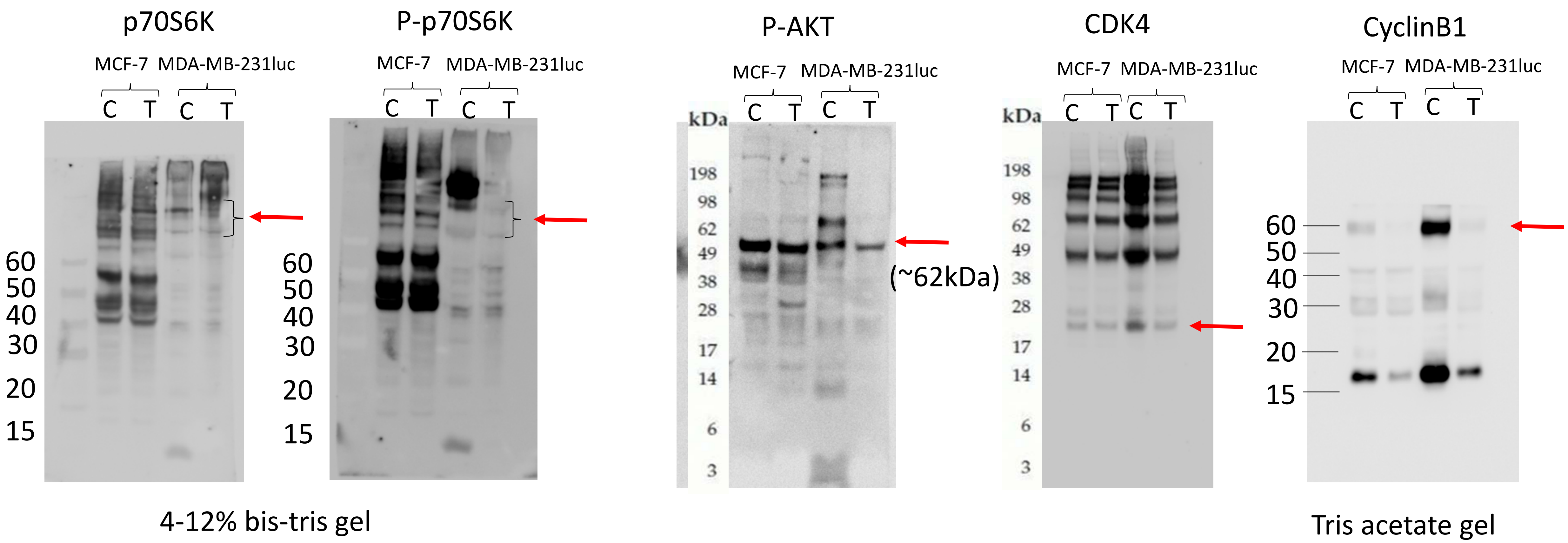

Supplemental Data 20.

Supplemental data for Figure 8. Effect of Rlip-LNA on signaling proteins of tumors from MCF7 and MDA-MB-231luc cell line xenograft. Representative unprocessed western blots of control (C) and Rlip-LNA (T) treated animal tumors as shown in figure 8. Red Arrow indicate cropped area included in figure 8.

C=CAS

T=Rlip-LNA

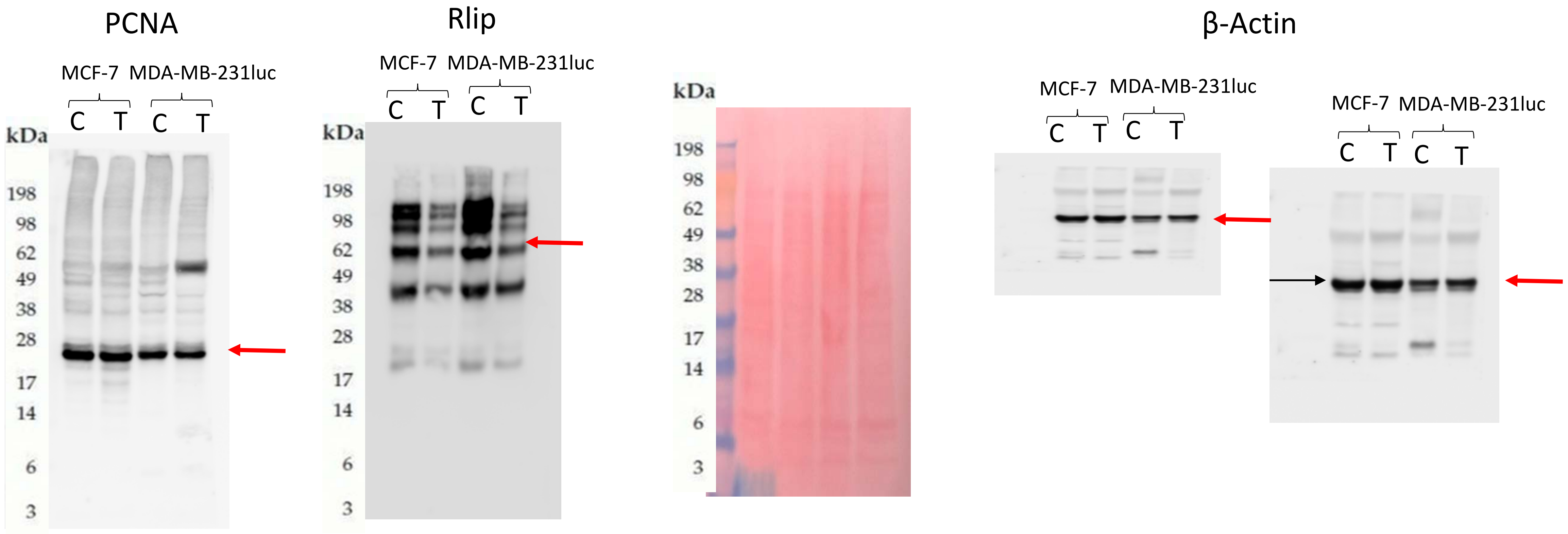

4-12% bis-tris gel

Representative Ponceau stains for the blots to visualize the transferred proteins confirms equal loading
