# Supplementary material for: Rlip Depletion Suppresses Growth of Breast Cancer
Source: Cancers (Basel). 2020 Jun 2;12(6):1446. doi: 10.3390/cancers12061446 (PMC7352702; doi:10.3390/cancers12061446)
Supplement: Supplementary file 1 [file cancers-12-01446-s001.zip › cancers-801743-suppl figures.docx]

Supplementary Materials: Rlip Depletion Suppresses Growth of Breast Cancer


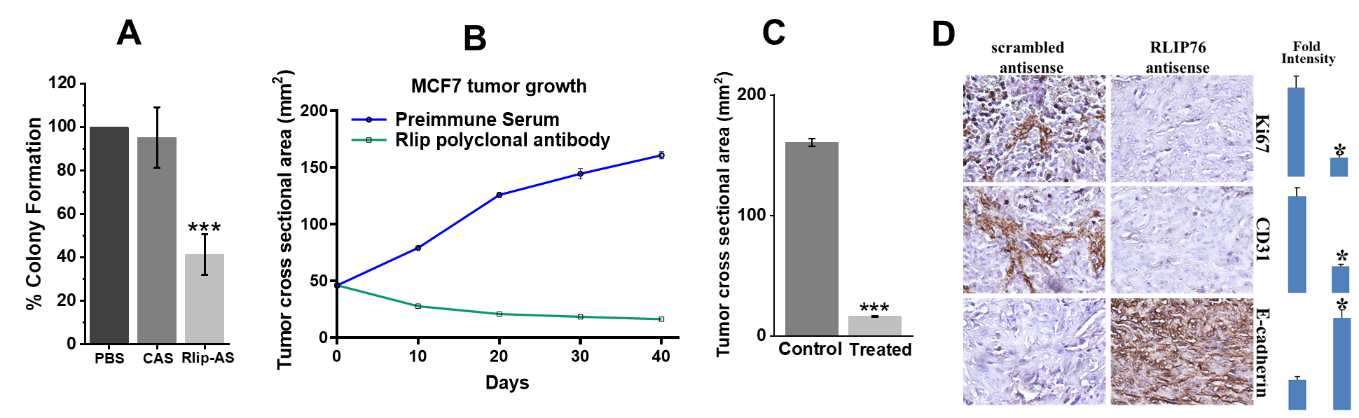


**Figure S1.** (**A**) Colony-forming assay for cell survival was performed with MCF7 cells per the Materials and Methods, with survival measured at 10 days after treatment. (**B**,**C**) Targeting Rlip with antibodies regressed MCF7 breast tumor growth in vivo. MCF7 cells were implanted subcutaneously on one flank of Hsd:athymic nu/nu female mice 5 days after subcutaneous implantation of estrogen pellets. Mice were treated with 200 μg of Rlip antibodies or with 200 μg/100 μL of pre-immune serum. Treatment was started when the cross-sectional area of the tumor was >42 mm^2^. (**D**) Immunostains of MCF7 tumor tissue against Ki67, CD31, and E-cadherin as well as the corresponding quantified relative staining intensity.


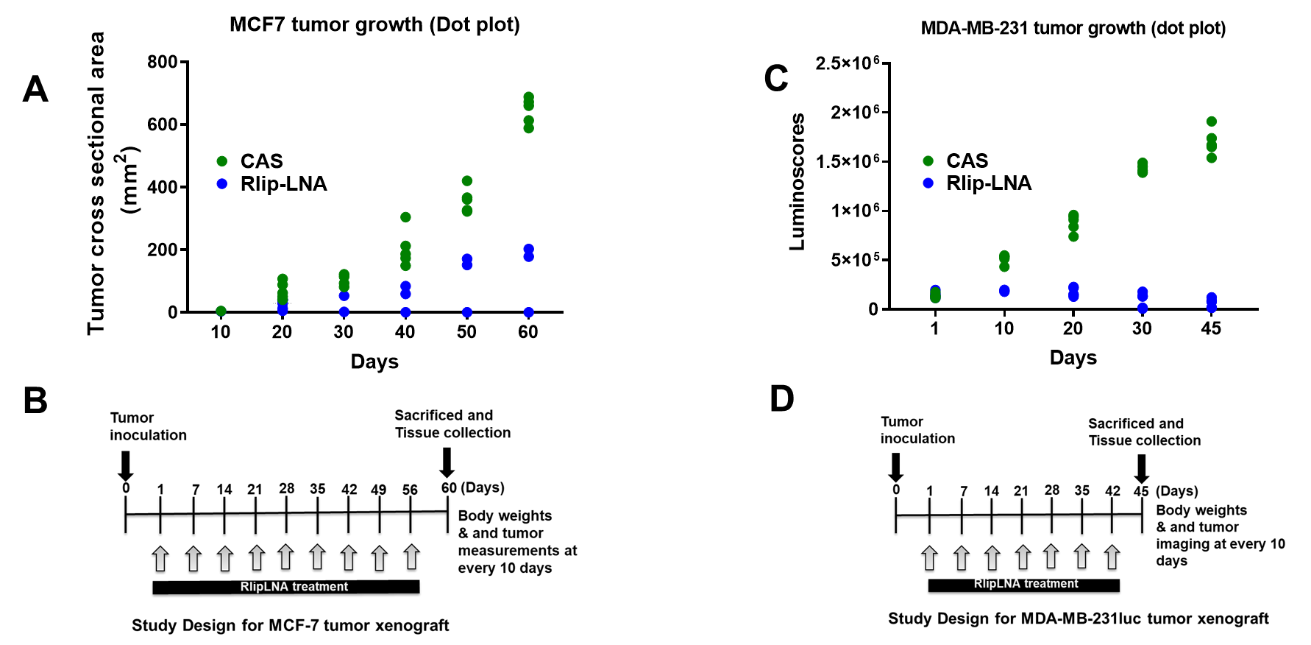


**Figure S2**. (**A,B**) Dot plot of tumor growth and study design of MCF7 xenograft. (**C**,**D**) Dot plot of tumor growth and study design of MDA-MB-231luc xenograft.


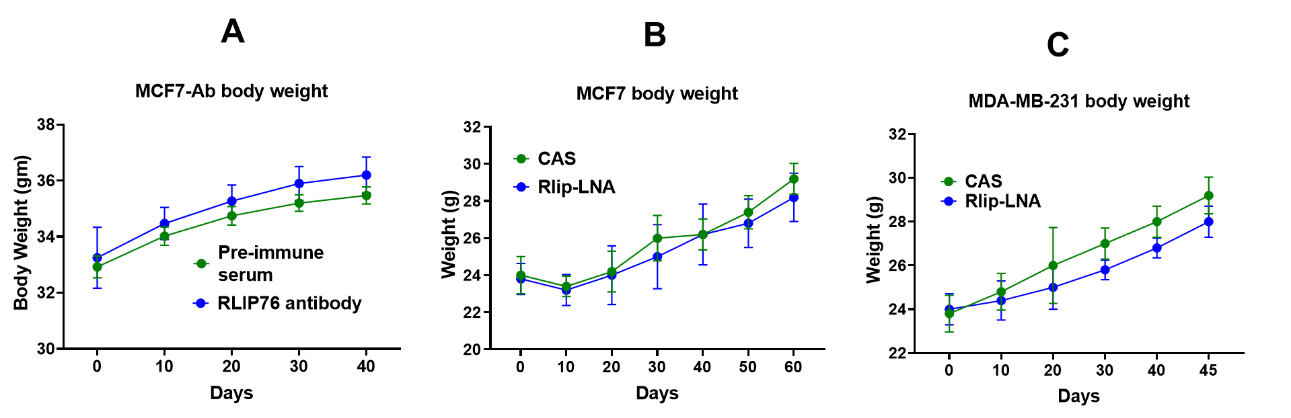


**Figure S3.** The effect of Rlip depletion on body weight change in mice bearing MCF7 or MDA-MB-231 tumor xenograft. (**A**) Treatment of MCF7 xenograft with pre-immune or Rlip antibody. (**B**) Treatment of MCF7 xenograft with CAS and Rlip-LNA. (**C**) Treatment of MDA-MB-231 xenograft with CAS or Rlip-LNA.
